# Small EV in plasma of triple negative breast cancer patients induce intrinsic apoptosis in activated T cells

**DOI:** 10.1038/s42003-023-05169-3

**Published:** 2023-08-04

**Authors:** Sujan Kumar Mondal, Derick Haas, Jie Han, Theresa L. Whiteside

**Affiliations:** 1 Department of Pathology, University of Pittsburgh School of Medicine and UPMC Hillman Cancer Center, Pittsburgh, PA 15213 USA; 2 Departments of Immunology and Otolaryngology, University of Pittsburgh School of Medicine, Pittsburgh, PA 15261 USA

**Keywords:** Breast cancer, Diagnostic markers

## Abstract

Small extracellular vesicles (sEV) in TNBC patients’ plasma promote T cell dysfunction and tumor progression. Here we show that tumor cell-derived exosomes (TEX) carrying surface PDL-1, PD-1, Fas, FasL, TRAIL, CTLA-4 and TGF-β1 induce apoptosis of CD8^+^T and CD4^+^T cells but spare B and NK cells. Inhibitors blocking TEX-induce receptor/ligand signals and TEX pretreatments with proteinase K or heat fail to prevent T cell apoptosis. Cytochalasin D, Dynosore or Pit Stop 2, partly inhibit TEX uptake but do not prevent T cell apoptosis. TEX entry into T cells induces cytochrome C and Smac release from mitochondria and caspase-3 and PARP cleavage in the cytosol. Expression of survival proteins is reduced in T cells undergoing apoptosis. Independently of external death receptor signaling, TEX entry into T cells induces mitochondrial stress, initiating relentless intrinsic apoptosis, which is responsible for death of activated T cells in the tumor-bearing hosts. The abundance of TEX in cancer plasma represents a danger for adoptively transferred T cells, limiting their therapeutic potential.

## Abstract (web summary)

Tumor cell-derived exosomes from triple negative breast cancer cells that are taken up by recipient T cells induce intrinsic apoptosis, leading to cell death, with implications for adoptive cancer immunotherapy.

## Introduction

Breast Cancer (BC) is the most common malignancy in women and is the leading cause of cancer deaths in women worldwide^1^. In 2018, more than two million women were newly diagnosed with BC worldwide^2^. Despite substantial recent progress in diagnosis and treatment, BC remains a therapeutic challenge due to its high invasion rate, metastasis, drug resistance and a frequent recurrence rate. BC is also a heterogenous disease, with triple negative breast cancer (TNBC) representing an especially aggressive subtype that is difficult to treat because tumor cells lack the three receptors: estrogen receptor, progesterone receptor and the epidermal growth factor receptor^3^. Expression levels of these receptors on tumor cells are used to guide therapy, and they correlate with prognosis^3^. TNBCs lacking these hormone receptors are difficult to treat and have a significantly increased rate of mortality^4^. There is an urgent need for the development and validation of predictive and prognostic biomarkers for TNBC^5^. The emergence of extracellular vesicles (EVs), as a potential non-invasive liquid tumor biopsy has captured attention of the scientific and medical communities, leading to increased interest in tumor-derived vesicles and their role in TNBC progression^6^.

Emerging data indicate that a subset of circulating small extracellular vesicles (sEV), also known as exosomes, has the potential to serve as non-invasive biomarkers of the disease presence, progression and response to therapy in cancer and other diseases^7–9^. sEV in body fluids, including plasma from patients with TNBC^10^, are viewed as a liquid tumor biopsy, because their molecular and genetic content reflects that of breast cancer cells producing the vesicles. Importantly, sEV can be serially and noninvasively harvested from body fluids at baseline and during follow up evaluations. These attributes have created an interest in evaluating sEV components (proteins, lipids, mRNA, miRNA) as potential biomarkers for breast cancer diagnosis, prognosis, and outcome^11^.

As all cells produce and release vesicles, EVs in body fluids are heterogeneous populations of differently sized vesicles with distinct cellular origins^12^. Among these vesicles, tumor cell-derived sEV or (TEX) have received special attention, and their molecular and functional characteristics have been extensively examined^13^. TEX isolated from supernatants of cancer cell lines or from plasma of cancer patients carry on their surface and in the vesicle lumen a large coterie of inhibitory receptor/ligands and have been shown to suppress functions of immune cells in vitro and in vivo^14,15^. In cancer, TEX production is increased^13^, and TEX account for a large but variable fraction of total sEV isolated from plasma by size exclusion chromatography (SEC), representing, e.g., from 20–70% of total sEV in melanoma^16^. However, total plasma sEV are a mix of vesicles derived from malignant and non-malignant cells; thus, they cannot be viewed as surrogates of TEX, and further fractionation by immune capture^17^ or other enrichment methods based on TEX molecular and functional profiles is necessary to separate TEX from total sEV in plasma. We and others have reported that sEV from plasma of patients with cancer, including TNBC, carry checkpoint proteins similar to those present on the tumor cell surface and, upon interaction with activated T cells, inhibit functions of the recipient cells and induce apoptosis^11,16,17^. As previously shown, this apoptosis results in DNA fragmentation in ~20% of T cells co-incubated with TEX^18^.

Mechanisms of immune cell death induced by signaling of death receptors and their cognate ligands have been extensively examined, and recent data suggest that their activation may lead either to cell death or to inflammation depending on the local milieu^19,20^. Fas and TRAIL receptors are best known for the ability to initiate apoptosis (programmed cell death) in the context of peripheral lymphocyte deletion or CTL-mediated killing^21,22^. However, early studies of these receptors in tissue or tumor cells showed that they can also promote NFκB activation and thus can drive inflammation^20^. It is not known whether the same molecular mechanisms are used by TEX which induce apoptosis of activated T cells.

We previously reported that apoptosis of CD8^+^ effector T cells by TEX positively correlated with disease stage, activity, and cancer progression^23^. This finding suggested that silencing of TEX-mediated apoptosis might benefit cancer patients. Here, we investigated the mechanisms responsible for TEX signaling, uptake and apoptosis of recipient T cells with the objective of blocking apoptosis induced by the death receptor/ligands on the surface of TEX produced by breast cancer cells. Surprisingly, we found that blocking of surface receptor/ligands on TEX interacting with T cells did not stop apoptosis in the recipient T cells. We report that TEX taken up by activated T cells induce intrinsic apoptosis, which irrevocably leads to cell death. Thus, therapeutic restraining of TEX from inducing death of CD8^+^ T cells by Ab blocking of the death receptor/ligands mediating external apoptosis is unlikely to work. Our data are highly relevant to adoptive cancer immunotherapy with activated T cells, which are in danger of being eliminated by TEX upon adoptive transfers into tumor-bearing hosts.

## Results

### Characterization of TEX produced by TNBC cell lines

**Fig. 1:**
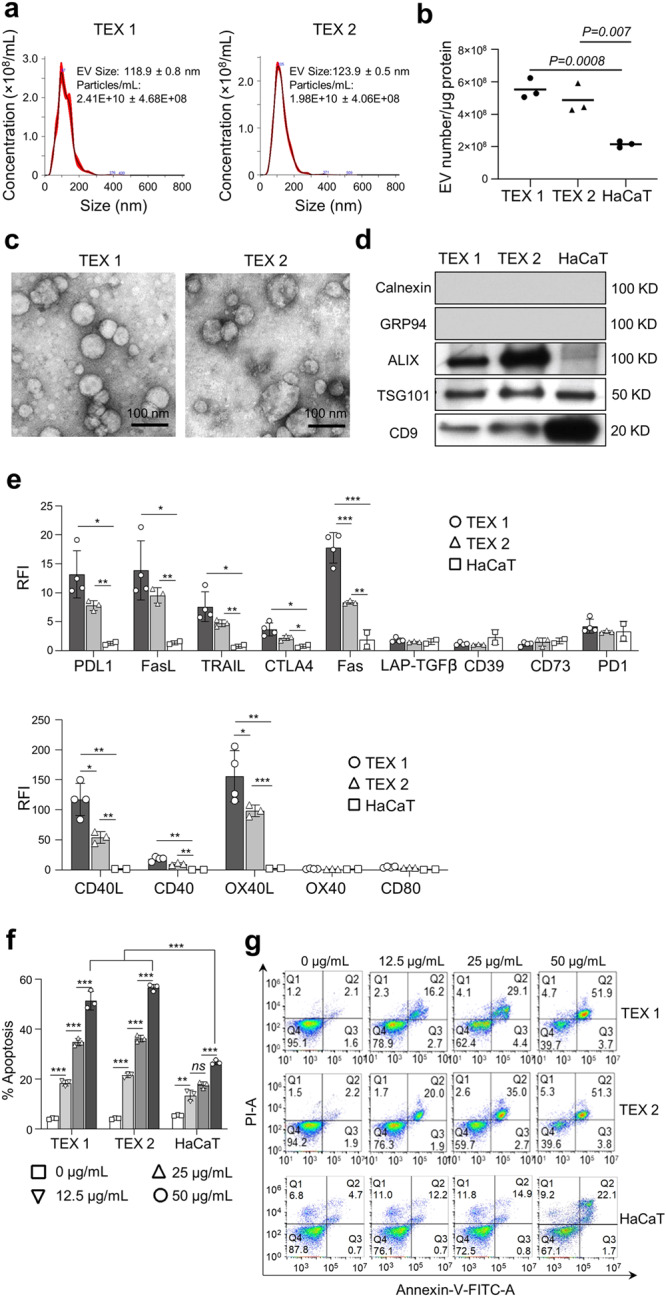
Characteristics of TEX1 isolated from supernatants of MDA-MB-231, TEX2 isolated from supernatants of MDA-MB-436 and sEV isolated from supernatants of non-malignant HaCat cell line. **a** Representative particle distribution images (NTA). **b** Ratios of TEX numbers/µg protein in SEC fractions #F4. **c** Representative TEM images; scale bar = 100 nm. **d** Western blot analysis. **e** On-bead flow cytometry analysis of immunosuppressive proteins on the sEV surface (upper row) and for immunostimulatory proteins (lower row). Data are presented as relative fluorescence intensity (RFI) values (means ± SD) obtained in three independent experiments. Unpaired *T*-tests were used to evaluate differences between sEV from different cell lines. Asterisks indicate p values: *p < 0.05; **p < 0.01; ***p < 0.001. **f** Apoptosis of CD8+ Jurkat cells co-incubated with increasing concentrations of TEX1, TEX2 or HaCaT sEV. Data are presented as means ± SD from 3 independent experiments. Data were analyzed by unpaired *t*-test. Asterisks indicate *p*-values: **p < 0.001; **p < 0.01, ns = no significant difference. **g** Representative flow cytometry images of apoptosis induced in Jurkat cells by increasing levels of TEX1, TEX2 or HaCaT sEV.

The vesicles isolated from supernatants of MDA-MB-231 and MDA-MB-436 TNBC cell lines (CL1 and CL2, respectively) were evaluated for the protein content, vesicle morphology (TEM), vesicle size (NTA) and the selected protein cargo by western blots (Fig. 1a–d). The particle number/protein ratios of recovered vesicles ranged from 5 × 10^8^ to 7 × 10^8^/µg protein. These vesicles measured 115–125 nm in diameter, contained CD9, TSG101 and ALIX, but did not carry cytoplasmic proteins (Grp94 and Calnexin) and showed vesicular morphology in TEM. According to the ISEV criteria for EVs nomenclature, they were classified as small EVs (sEV) or TEX^24^. sEV isolated from the supernatants of non-malignant HaCaT cells had similar characteristics (Fig. 1).

TEX produced by the TNBC-CLs and HaCaT-derived sEV were evaluated for the presence of surface immunoregulatory proteins by on-bead flow cytometry. The representative results for surface expression levels of individual proteins expressed as relative fluorescence intensity (RFI) values for TEX1, TEX2 and HaCaT sEV are shown in Supplementary Fig. 1a–c. The phenotypic characteristics of TEX1, TEX2 and sEV from HaCaT are summarized in Fig. 1e. TEX1 had significantly higher levels of PDL-1, Fas, FasL, TRAIL and CTLA4 as well as CD40L, CD40 and OX40L surface proteins than TEX2. Both TEX1 and TEX2 had significantly higher expression levels of these inhibitory and stimulatory proteins relative to sEV from HaCaT cells. In contrast, expression levels of TGF-β, CD39, CD73, PD-1, OX40 and CD80 were comparable in sEV from cancer and non-malignant cells.

Functional activity of TEX1, TEX2 and HaCaT sEV was measured in Annexin V/PI binding assays, and results in Fig. 1f, g show that apoptosis induced by these vesicles in CD8^+^ Jurkat T cells was dependent on the vesicle concentration. At the highest TEX concentrations tested (50 µg/mL), apoptosis of Jurkat cells approached 60% while apoptosis mediated by HaCaT sEV was below 30% (p < 0.001). The data suggest that although apoptosis of non-malignant cells mediated by sEV was detectable even at the lowest sEV concentration (12.2 µg/mL), it was consistently significantly lower than apoptosis mediated by TEX1 or TEX2 (Fig. 1f**)**. Although TEX1 carried higher levels of pro-apoptotic FasL and TRAIL than TEX2 (Fig. 1e), TEX1 and TEX2 induced comparable levels of apoptosis in Jurkat target cells.

### Characterization of sEV isolated from plasma of TNBC patients

**Fig. 2:**
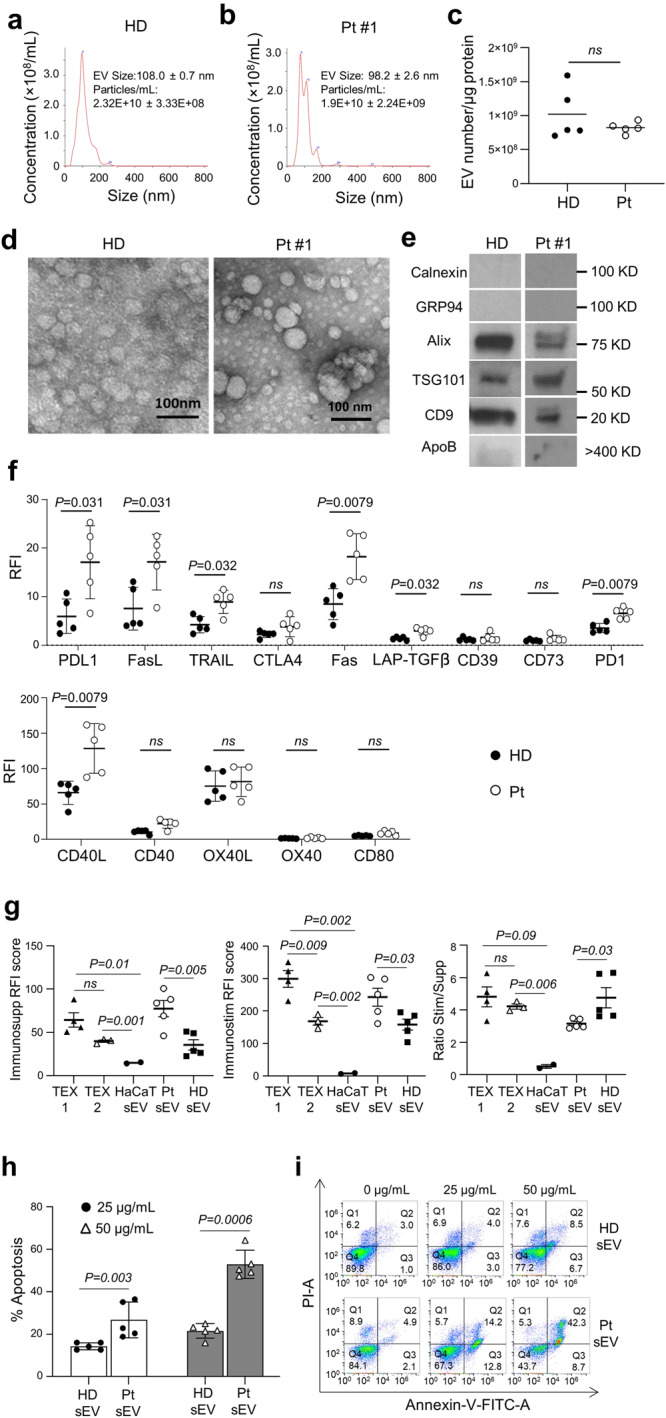
Characterization of sEV isolated from plasma of TNBC-patients or healthy donors (HDs). **a**, **b** Representative particle distribution images (NTA) of sEV derived from plasma of a HD and TNBC Pt #1. **c** sEV numbers/µg protein in fraction #4 for Pts and HDs. **d** Representative TEM images of sEV from plasma of a HD and TNBC-Pt #1. **e** Western blots of sEV (fraction #4) isolated from plasma of a HD and TNBC-Pt #1. **f** On bead flow cytometry of sEV from plasma of TNBC Pts and HDs. Shown are RFI values for immunosuppressive proteins on the sEV surface (upper row) and for immunostimulatory proteins (lower row). Data are presented as means ± SD. Mann-Whitney tests were used to evaluate differences between TNBC Pts and HDs. ns = no significant difference. **g** The RFI scores for immunosuppressive or immunostimulatory proteins and the ratios of stim/supp proteins calculated for all the above listed sEV as described in the text. Note the higher suppressive and stimulatory scores for TNBC Pt’s sEV than for HD’s sEV and the significantly higher stim/supp ratio for sEV of HDs than for TNBC Pts. **h** Apoptosis (%) induced in activated primary human CD8 + T cells by sEV from plasma of HDs (*n* = 5) and TNBC-Pts (*n* = 5). Data were analyzed by an unpaired *t*-test and are presented as means ± SD. **i** Flow cytometry images of apoptosis induced in CD8 + T cells by increasing doses of sEV from a representative HD or TNBC Pt.

The sEV isolated from plasma of patients with metastatic TNBC and from plasma of HDs were also characterized for their size, numbers, endocytic origin, morphology, the surface phenotype, and apoptotic activity (Fig. 2a–c). By NTA, the sEV isolated from TNBC Pts’ plasma were somewhat smaller in size (mean = 99 nm) compared to HDs’ vesicles (mean = 109 nm) and were more heterogenous, as also indicated by their vesicular morphology (Fig. 2d): by TEM, their diameter ranged from 30–150 nm. The particle number/protein ratios for vesicles from HD’s or TNBC Pts’ plasma were similar, ranging from 5×10^8^ to 1×10^9^/µg protein. Plasma derived sEV carried CD9, TSG101 and ALIX, but did not carry cytoplasmic proteins (Grp94 and calnexin) and contained minimal ApoB (Fig. 2e).

The phenotypic characteristics of sEV from plasma of TNBC Pts and HDs are shown in Fig. 2f with representative RFIs illustrated in Supplementary Fig. 2. As shown, several checkpoint proteins (PD-L1, Fas L, TRAIL, Fas, TGF-β and PD-1) were significantly upregulated in sEV isolated from plasma of TNBC Pts. Among the immunostimulatory proteins, only CD40L was significantly upregulated on TNBC sEV vs HD sEV (Fig. 2f). Having determined the RFI values for each immunoregulatory protein carried by the vesicles, we summarized the RFIs for suppressive and stimulatory proteins carried by sEV of each donor (see Supplementary Tables 1 and 2). We calculated the RFI and immunosuppressive as well as immunostimulatory scores for sEV of TNBC Pts and HDs as previously described^16^ (Supplementary Tables 1 and 2**)**. These suppressive and stimulatory scores as well as the stim/supp ratios for TEX1, TEX2, and sEV shown in Fig. 2g indicate that: (i) the mean suppressor scores are not different for TEX1 and TEX2 but differ from that for HaCaT sEV (*p* = 0.01 and *p* = 0.001, respectively). The mean suppressor score for TNBC Pts’ sEV is higher than that for HD’s sEV (*p* = 0.005); (ii) the mean stimulatory score is higher for TEX1 than TEX 2 (*p* = 0.009), and the mean scores for both are higher than that for HaCaT sEV (*p* = 0.002). The mean stimulatory score for TNBC Pts’ sEV is higher than that for HD’s sEV (*p* = 0.03); and (iii) the mean stim/supp ratio is not different for TEX1 and TEX 2, and the ratios for both are higher than that for HaCaT sEV For TNBC Pts’ sEV, the mean ratio of ~2.5 is lower than that of sEV from HDs at ~5.0 (*p* = 0.03). In aggregate, this phenotypic analysis indicates that the immunostimulatory and immunosuppressive protein profiles of sEV in TEX differ from those of sEV produced by non-malignant cells; further, the sEV profiles for TNBC Pts differ from those of HDs’ sEV.

Apoptotic activity of sEV from plasma of TNBC Pts was compared to that mediated by HD sEV using primary human CD8^+^ T cells as targets. The data in Fig. 2h, i show that apoptosis of activated human primary CD8^+^ T cells mediated by TNBC Pts’ sEV is concentration dependent and significantly exceeds that mediated by sEV of HDs.

Overall, the data obtained with TEX and with plasma-derived sEV of TNBC Pts suggest that these vesicles are armed to mediate T cell death. In contrast, sEV obtained from cultured non-malignant cells or from plasma of HDs carry fewer checkpoint proteins and mediate significantly lower apoptosis. The data are consistent with our previous reports that the ability to induce T cell apoptosis is a property of TEX and not of sEV produced by non-malignant cells.

### Susceptibility of immune cells to TEX-mediated apoptosis

To determine whether TEX-mediated apoptosis was limited to activated CD8^+^ T cells or extended to CD4^+^ T cells and other subsets of immune cells, we co-incubated various immune cells with TEX. Because TEX produced by TNBC CLs are readily available in large numbers, they served as a useful surrogate model of interactions between sEV and human T cells in the studies described below.

**Fig. 3:**
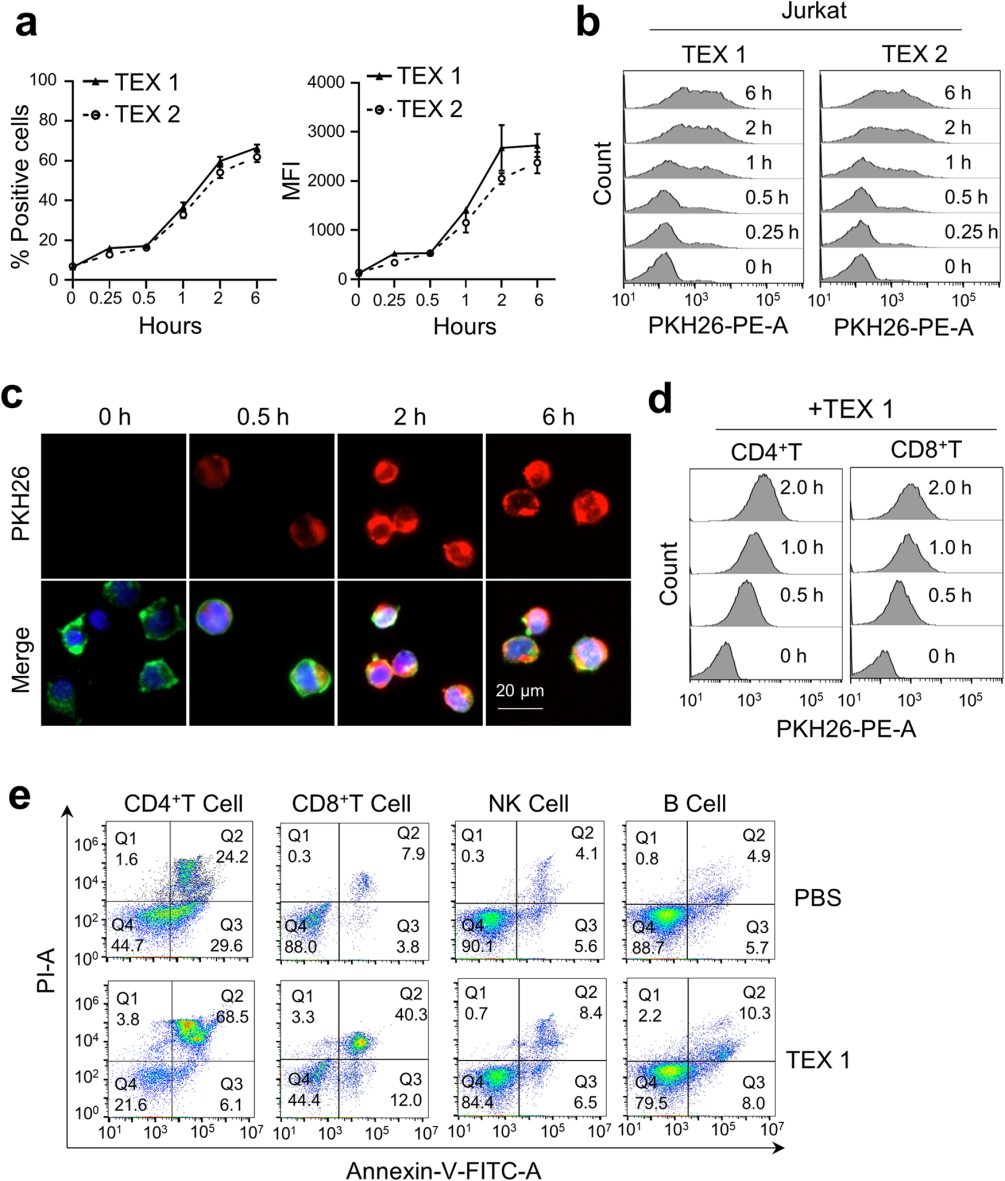
Uptake of TEX labeled with PKH26 by immune cells. TEX isolated from supernatants of TNBC cell lines were labeled with the PKH26 dye and were co-incubated with recipient immune cells for various time periods. **a** Percentages of CD8+ Jurkat cells up-taking labeled TEX during coincubation as measured by flow cytometry. Data are mean values from two independent assays and are shown in the excel table in Supplementary Data 1. **b** Representative histograms for the uptake of labeled TEX presented as variables of the uptake time. **c** Microscopic images of CD8+ Jurkat cells co-incubated with PKH26 labeled TEX1. TEX = Red, Actin = green, DAPI stained nuclei = blue; scale bar = 20 µm. **d** Comparison of TEX1 uptake by activated primary CD4 + T and CD8 + T cells at different time points. **e** Flow cytometry images of apoptosis induced in activated primary CD4 + T, CD8 + T, B and NK cells following co-incubation with TEX1.

First, TEX were labeled with the PKH26 dye and extensively washed to remove PKH26 dye micelles^25^. Immune cells (CD8^+^ Jurkat T cells, primary CD4^+^ or CD8^+^ T cells) were co-incubated with labeled TEX, and the uptake of PKH26-labeled TEX by the recipient cells was monitored by fluorescence microscopy and flow cytometry (Fig. 3a–c). We observed that little or no uptake of labeled TEX by CD8^+^ T cells occurred during the first 15–30 min of co-incubation. After 6 h of co-incubation, ~ 60% of CD8^+^ T cells contained labeled TEX. The uptake profiles of activated primary CD4^+^ and CD8^+^ T cells shown in Fig. 3d resemble each other and are comparable to those obtained with Jurkat T cells. These uptake profiles consistently indicate there is an early delay in TEX uptake by activated T cells. This finding confirms our earlier observations of relatively slow TEX uptake by human primary CD8^+^ and CD4^+^T cells relative to NK cells, B cells or monocytes, which rapidly internalized TEX^26^. Thus, among various recipient immune cells tested, CD4^+^ and CD8^+^ T cells internalized TEX at the slowest uptake rate^26^. This prolonged TEX contact with the surface of recipient T cells likely allows for the receptor/ligand type of interactions to occur prior to TEX internalization.

We reported above that co-incubation of TEX with CD8^+^ T cells resulted in their apoptosis. Since there was no difference in uptake of TEX by recipient CD4^+^ and CD8^+^ T cells, we expected that TEX also induce apoptosis of CD4^+^ T cells. Next, TEX-induced apoptosis of primary human CD4^+^, CD8^+^, B and NK cells was evaluated. The data in Fig. 3e show results of TEX co-incubation (at the protein concentration of 50 µg/mL for 6 h) with primary activated CD8^+^ T cells (~52% apoptosis), activated CD4^+^ T cells (~30% apoptosis above the background of ~50% apoptosis with PBS), and activated human B cells or NK cells (13% and 18% apoptosis, respectively). Similar results were also previously reported by us^17^. Consistently, co-incubation of TEX at 50 µg/mL with CD8^+^ Jurkat T cells or with activated CD8^+^ primary human T cells for 6 h induced concentration-dependent apoptosis in ~50% of recipient T cells. In initial experiments, we also measured downregulation of CD69 expression levels in T cells co-incubated for 24 h with TEX (5–20 µg/mL) by flow cytometry. A reduction of 30–50% in CD69 expression levels was observed in T cells relative to PBS controls (n = 3) Results of TUNEL assays with CD8+Jurkat cells as recipients also indicated that TEX induce DNA fragmentation in T cells, as we previously reported^18^.

### TEX induced apoptosis of activated T cells reflects complementary receptor/ligand signaling

**Fig. 4:**
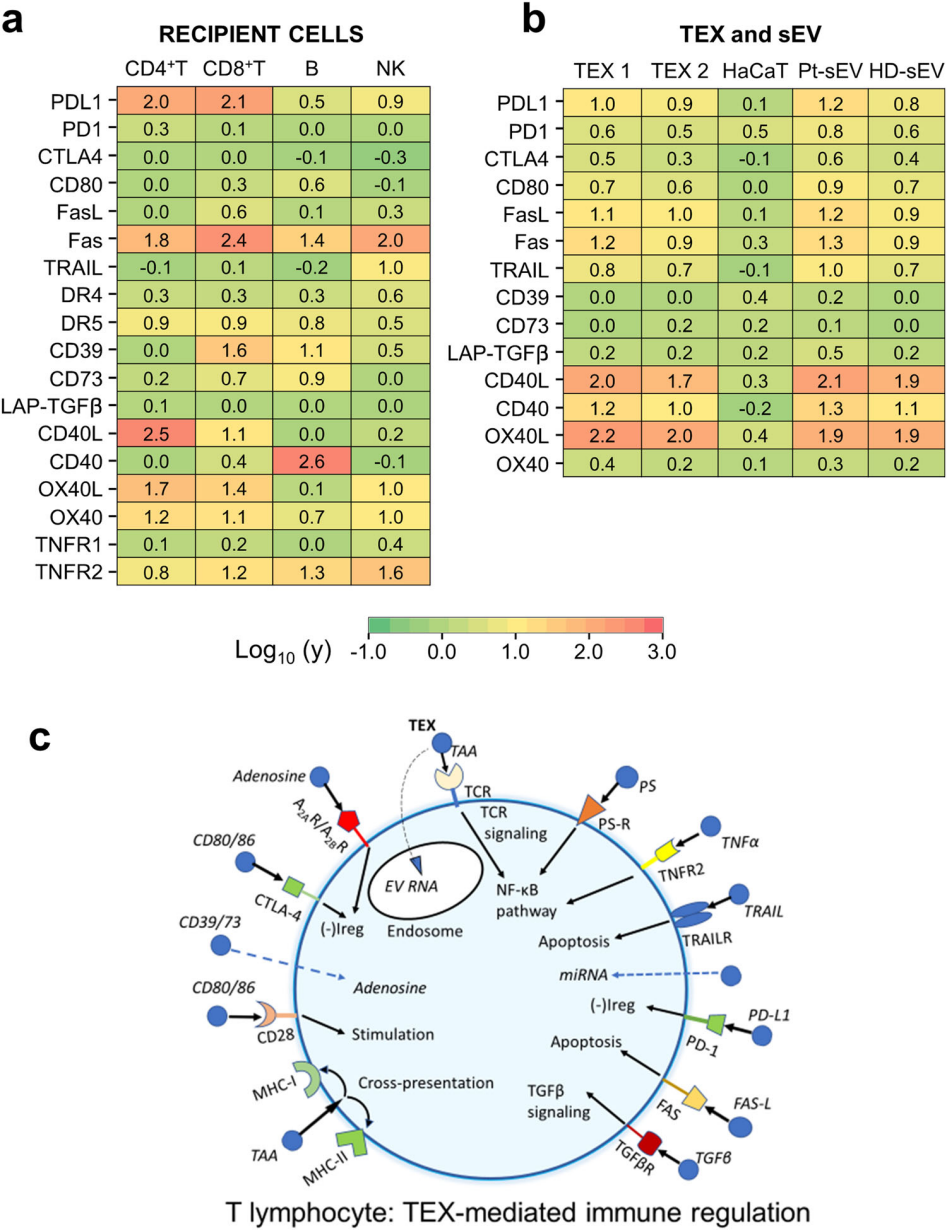
Immunoregulatory proteins expressed on the surface of recipient immune cells and on TEX or sEV of malignant and non-malignant origins. **a** The heatmap presenting mean RFI values for proteins expressed on the surface of activated primary CD4 + T, CD8 + T, B and NK cells. **b** The heatmap presenting mean RFI values for proteins found on the surface of TEX1, TEX2, HaCaT sEV, TNBC Pt-sEV and HD-sEV. **c** An image of a T cell interacting with various TEX (shown as blue vesicles) that carry immunoregulatory proteins on the surface membrane. TEX binding to complementary receptors expressed by the T cell initiate immunoregulatory signals which result in immune downregulation [(-)Ireg] and/or immune stimulation. The sum of these simultaneously delivered signals will determine whether TEX mediate immune suppression or immune stimulation in a recipient T cell. Note that a single sEV might carry multiple signaling proteins on its surface membrane.

Phenotypic profiles of immunoregulatory surface proteins in activated primary CD4^+^ and CD8^+^ T cells (Supplementary Fig. 3a, b; Fig. 4a) as well as B cells and NK cells (Fig. 4a) confirm the presence of receptors and/or ligands able to interact with sEV. The heatmap in Fig. 4a shows differential expression levels of various signaling receptors (PD1, CTLA4, Fas, CD39, CD73, CD40, OX40, TNFR, DR4, DR5) and—ligands (PD-L1, CD80, FasL, TRAIL, CD40L, OX40L) on the surface of recipient immune cells potentially able to interact with TEX in co-incubation assays. Expression levels of these receptors on the cell surface might determine relative sensitivity of activated CD8^+^T cells and resistance of B cells and NK cells to TEX-induced apoptosis. Specifically, higher expression levels of PD-L1, Fas and of stimulatory receptor/ligands, CD40L OX40L and OX40, were seen in CD4^+^ T cells, while activated CD8^+^ T cells expressed higher levels of PD-L1, Fas, CD39, CD73 and somewhat lower levels of OX40 and OX40L. Relative to CD8^+^ T cells, B cells expressed lower levels of inhibitory receptor/ligands and were enriched in CD40, OX40 and CD80 costimulatory proteins. NK cells expressed moderate levels of PD-L-1, high levels of Fas and TNFR2 but relatively lower levels of the other death receptor/ligands (Fig. 4a).

The heatmap in Fig. 4b presents the mean RFI values of immunoregulatory proteins detected on the surface of TEX1, TEX2, and sEV of TNBC Pts, HD’s and HaCaT cells. The low expression levels of all the receptor/ligands in HaCat sEV is evident. In sEV of TNBC patients, HDs and TEX1/TEX2 we see relatively moderate expression levels of all the suppressive proteins and significantly elevated expression levels of CD40L, CD40 and OX40L. The enrichment of these co-stimulatory proteins in all sEV is consistent with the vesicle ability to interact with and activate recipient cells. In aggregate, this phenotypic analysis of sEV indicated a moderate enrichment in immunoregulatory proteins in TNBC Pts sEV relative to HD’s sEV. An underlying assumption of these aggregate results is that signaling of the complementary receptor/ligands present on the surface of recipient T cells and carried by TEX drives T cell apoptosis in patients with cancer, as indicated in Fig. 4c. It appears that an activated CD8 + T cell surrounded by numerous sEV in the circulation of a TNBC patient might simultaneously receive many different regulatory signals that determine its fate.

### Blocking of death receptor/ligand signaling fails to prevent TEX-induced T cell apoptosis

**Fig. 5:**
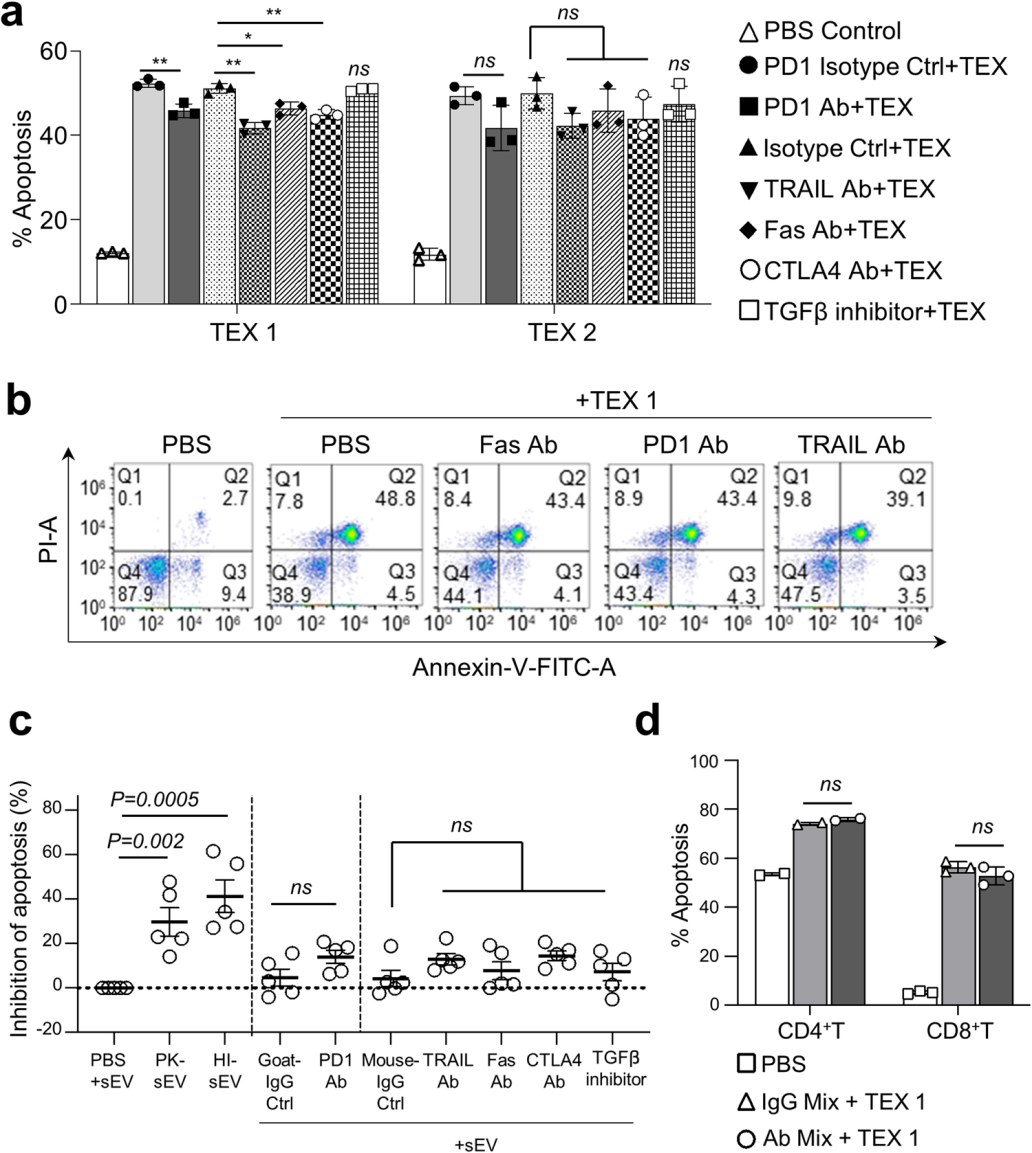
TEX-induced apoptosis of activated human CD8 + T cells is not blocked by neutralizing Abs or specific inhibitors. **a** Apoptosis (%) induced in CD8 + T cells by TEX1 or TEX2 (50 µg/mL) was not significantly blocked by the neutralizing Abs used or the TGF-β inhibitor. **b** Representative flow cytometry for apoptosis of activated CD8 + T cells co-incubated with TEX1 in the presence of blocking Abs. **c** Inhibition of apoptosis by various neutralizing Abs of activated CD8 + T cells co-incubated with sEV from TNBC Pts (25 µg/mL). The pretreatment of TNBC-pts sEV with protein kinase (PK) or heat (HI) significantly reduced but did not eliminate T cell apoptosis. **d** The pretreatment of CD8 + T cells or activated primary CD4 + T cells with the mix of blocking Abs specific for PD1, TRAIL, Fas and anti-CTLA4 (used at the f.c. of 10, 10, 10 and 20 µg/mL, respectively) failed to reduce apoptosis induced by TEX 1 (50 µg/mL). In the blocking assays, primary human T cells were incubated with different blocking Abs and the TGF-β inhibitor. All Abs were used at the f.c. of 10 µg/mL, except for anti-CTLA4 Abs which were used at the f.c. of 20 µg/mL. TGF-β inhibitor was used at the f.c. of 50 nM. Blocking was performed for 30 min before co-incubation for 6 h with TEX or sEV from plasma of TNBC-Pts or HDs. Apoptosis was evaluated by Annexin-V binding assays. The data presented in (**a**) and (**d**) are means ± SD from 3 independent experiments.

TEX and sEV from TNBC Pts’ plasma carry various death-inducing receptors/ligands on the surface membrane (Fig. 4b). The recipient CD4^+^ and CD8^+^T cells express the complementary surface receptor/ligands (Fig. 4a). Thus, their co-incubation results in T cell apoptosis (Fig. 2h, i). However, when CD8^+^ Jurkat T cells were co-incubated with TEX1 or TEX2 (50 µg/mL) or TNBC sEV (25 µg/mL), the resulting apoptosis could not be blocked by neutralizing Abs specific for Fas, PD-1, CTLA 4 or TRAIL or the TGF-β pharmacologic inhibitor added to the reaction at varying concentrations (10–20 µg/mL) prior to or during the co-incubation (Fig. 5a). A mix of these neutralizing reagents failed to induce T cell apoptosis. Only minimal inhibition (NSD) of T cell apoptosis mediated by TEX ( ~ 10% of isotype control) was observed with Abs to PD1, TRAIL, Fas, FasL, CTLA4 or the TGF-β inhibitor (Fig. 5a, b). The same resistance to apoptosis was observed regardless of different inhibitor concentrations used or whether inhibitors were delivered prior to or during TEX co-incubation with vesicles. Notably, apoptosis of Jurkat T cells by the synthetic FasL or by sTRAIL was completely inhibited by the anti-Fas mAb or anti-TRAIL Abs, respectively, validating neutralizing activity of these Abs (Supplementary Fig. 4a, b). All blocking experiments were repeated x3 with each inhibitor, but T cell apoptosis by TEX could not be significantly inhibited. Further, when CD8+ Jurkat cells were incubated with anti-PD-1 (10 µg/mL) or anti-CTLA4 (20 µg/mL) Abs for 30 min prior to addition of CH11 Abs (10 ng/mL) or staurosporine (0.5 µM), apoptosis was not inhibited as indicated by the cleavage of caspase 8, caspase 3 and PARP (Supplementary Fig. 5).

The above-described results with Jurkat cells were reproduced when we attempted to block apoptosis of primary human T cells by sEV isolated from TNBC Pts’ plasma (Table 1).

**Table 1:** Apoptosis (%) induced by sEV isolated from TNBC patients’ plasma in activated primary human CD8^+^ T cells^#^.

Co-culture	Pt #1	Pt #2	Pt #3	Pt #4	Pt #5
T cell + PBS	8.2	8.2	8.2	6.3	7.3
T cell + sEV	20.4	18.8	22.8	35.1	36.4
T cell + PK-sEV	15.7	16.2	17.7	18.3	21.2
T cell + HI-sEV	13.4	13.7	16.5	13.5	16.0
T cell + Goat-IgG Ctrl + sEV	18.2	18.2	19.2	35.8	37.9
T cell + PD1 Ab + sEV	19.1	17.4	18.0	28.7	30.2
T cell + Mouse-IgG Ctrl + sEV	19.8	18.4	18.5	35.2	37.3
T cell + TRAIL Ab + sEV	18.4	17.3	17.7	30.7	32.1
T cell + Fas Ab + sEV	20.0	15.8	18.4	35.1	35.7
T cell + CTLA4 Ab + sEV	16.9	15.9	18.1	31.2	33.3
T cell + TGFβ inhibitor + sEV	20.1	16.9	19.0	36.9	31.7

^#^sEV were isolated from plasma of patients with TNBC as described in Materials and Methods. Primary human CD8+ T cells were isolated from PBMC of healthy donors. T cells were co-incubated with sEV (25 µg/mL) blocking antibodies or inhibitors (at concentrations indicated in Table 1) for 6 h, and T cell apoptosis was measured by Annexin V binding assays.

Apoptosis was not inhibited by any of the individually tested blocking agents used at the concentrations indicated above (Fig. 5c) or by a mix of all agents (Fig. 5d). In contrast to blocking mAbs, the pretreatment of sEV from TNBC Pts’ plasma with proteinase K (PK) reduced T cell apoptosis by ~30% of controls (range 10–50%) and heating of these vesicles at 80 °C for 1 h by ~40% (range 30–50 %). Heat or PK pretreatments of sEV only reduced but did not eliminate their ability to mediate apoptosis of primary human T cells.

In addition to TEX produced by TNBC-CLs, we tested TEX isolated from supernatants of other human tumor cell lines (e.g., Mel526 and PCI-13) for the capability to induce T cell apoptosis. After confirming the presence of the death receptor/ligands on the surface of TEX by on-bead flow cytometry, TEX were co-incubated with Jurkat T cells. Comparably to TEX from TNBC-CLs, melanoma cell-derived or HNSCC cell-derived TEX, but not HD’s sEV, induced strong concentration-dependent apoptosis in activated T cells, as also previously reported by us^16^. This apoptosis induced by TEX originating from various human tumor cells could not be completely inhibited by various blocking Abs or pharmacologic inhibitors interfering with the death receptor/ligand-mediated signaling initiated at the surface of T cells interacting with TEX.

As TEX produced by TNBC CLs carry MHC class I and class II molecules embedded in the surface membrane, we co-incubated human primary T cells with TEX in the presence of Abs specific for the MHC antigens. Blocking of the HLA epitopes on T cells or TEX had no effect on TEX-mediated cell death and did not alter apoptosis levels these TEX induced in activated T cells. These data further confirm that blocking of surface antigens involved in TEX-T cell interactions had no effect on TEX mediated apoptosis of activated T cells.

### Interference with TEX uptake by recipient T cells does not block T cell apoptosis

**Fig. 6:**
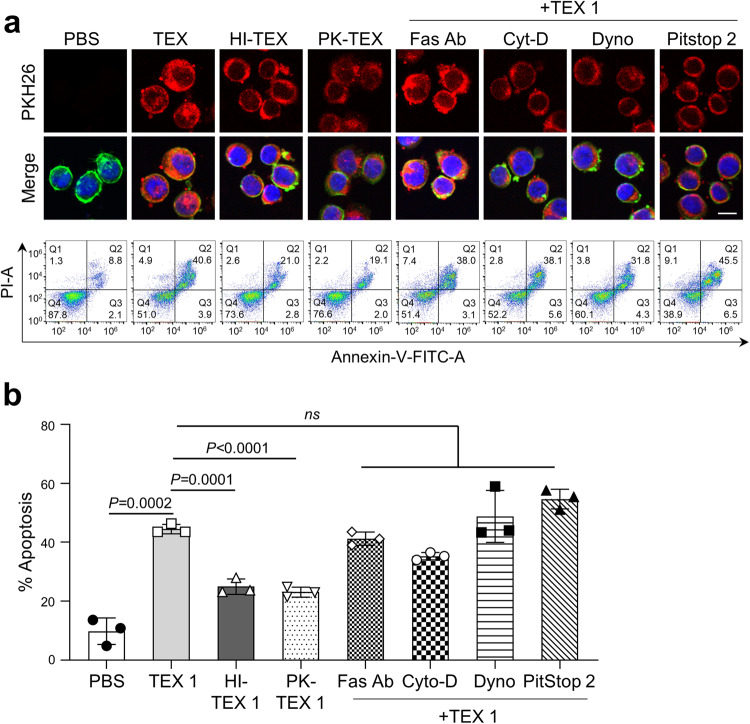
Inhibition of TEX uptake by T cells may reduce but does not eliminate apoptosis in recipient T cells. **a** Representative images of cellular uptake of PKH26-labeled TEX 1 by Jurkat T cells (upper row) and corresponding images of apoptosis induced by TEX 1 in the recipient T cells (lower row). TEX 1 labeled with the PKH26 dye were pretreated with protein kinase (PK;1 µg) or heat (HI; 80 °C for 1 h) and were co-incubated with CD8+Jurkat T cells for 2 h to measure vesicle uptake and for 6 h to measure T cell apoptosis. CD8+Jurkat T cells were also pre-incubated individually with anti-Fas antibody (10 µg/mL), Cytochalasin-D (20 µM), Dynosore (10 µM) or Pit Stop-2 (10 µM) for 30 min followed by 2 h coincubation with PKH26 labeled TEX 1 (50 µg/mL) to measure uptake and for additional 6 h to measure T cell apoptosis. Images were taken at 40x mag. TEX = Red, Actin = green, DAPI stained nuclei = blue; scale bar = 10 µm. To measure T cell apoptosis, Annexin V binding assays were performed following 6 h co-incubation with TEX 1. **b** Apoptosis (%) induced by TEX 1 co-incubated with CD8+Jurkat T cells following pretreatments of TEX or CD8+Jurkat cells with various blocking agents as described above. Data are means ± SD from 3 independent experiments. Data were analyzed by ANOVA followed by Dunnett’s post hoc analysis. ns = no significant difference.

To induce apoptosis in recipient cells, sEV must first deliver signals to the cell surface and/or enter recipient cells. We considered the possibility that blocking uptake of sEV by recipient T cells might reduce/prevent their apoptosis. To this end, we pretreated TEX with PK (1 µg PK/2.5 µg TEX protein) or heat (80 °C for 1 h) prior to co-incubation with T cells, expecting to eliminate signaling of surface proteins that might facilitate the TEX entry into T cells. However, neither PK nor heat eliminated the entry of PKH26-labeled TEX into T cells (Fig. 6a), although both reduced (*P* < 0.0001) T-cell apoptosis to levels of ~40–45% of controls (Fig. 6a, b). Increasing PK concentrations or the time of TEX pretreatment did not further reduce T cell apoptosis.

Next, we attempted to block TEX entry into T cells using Dynasore (10 µM) or PitStop 2 (10 µM), which inhibit clathrin-mediated endocytosis, or Cytochalasin D (20 µM), an inhibitor of micropinocytosis and phagocytosis. Figure 6a shows that like PK or heat pretreatment, these inhibitors reduced but did not prevent TEX uptake and entry into T cells. Analysis of images shown in Fig. 6a indicated that Fas Ab and PitStop 2 reduced TEX uptake by ~10% vs heat at ~20% and PK, Cytochalasin D or Dynasore at ~30%. Apoptosis of the recipient T cells was either not reduced at all (Pit Stop 2) or only mildly reduced (NSD) by Dynasore, Cytochalasin D or by neutralizing anti-Fas mAb (Fig. 6b). Thus, neither proteolytic digestion nor protein denaturation nor attempts at blocking the vesicle entry into T cells prevented apoptosis of T cells cross talking with TEX. Our data suggest that apoptotic signals delivered by sEV binding to T cells persistently drive apoptosis in the recipient T cells and cannot be arrested or eliminated by interfering with signaling of death receptors/ligands on the cell surface.

### TEX induced apoptosis is not prevented by caspase inhibition in T cells

**Fig. 7:**
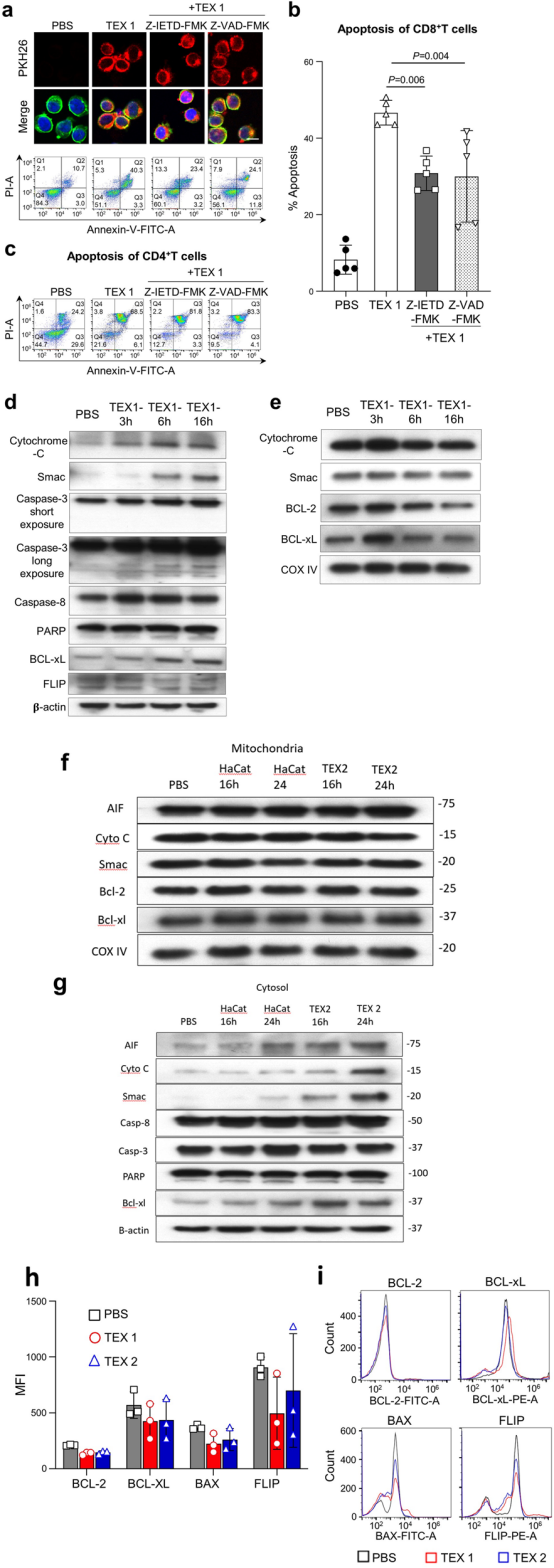
Effects of caspase inhibitors on TEX uptake by CD8+Jurkat cells and TEX-induced Jurkat cell intrinsic apoptosis. **a** Representative cellular uptake of TEX1 by CD8+Jurkat cells (upper panel) and resulting apoptosis (lower panel). Recipient Jurkat cells were pre-incubated with caspase 8 inhibitor (Z-IETD-FMK, 100 µM) or pan caspase inhibitor (Z-VAD-FMK, 100 µM) for 30 min prior to co-incubation with TEX1 for 2 h to measure TEX uptake by T cells. Then the T cells were incubated for additional 6 h to measure apoptosis. Cellular uptake images were acquired by confocal microscopy at ×40 mag. TEX = Red, Actin = green, DAPI stained nuclei = blue; scale bar = 10 µm. **b** Apoptosis (%) induced by TEX1 in CD8+Jurkat cells pretreated with caspase inhibitors as in (**a**) was significantly reduced but not eliminated. Data were analyzed by ANOVA followed by Dunnett’s post hoc analysis and represent means ± SD from 3 independent experiments. **c** Apoptosis (%) induced by TEX1 in activated CD4+T cells pre-incubated with caspase inhibitors. No reduction in CD4+ T cell apoptosis was noted. **d**, **e** Western blots showing expression of pro-apoptotic and anti-apoptotic proteins in the mitochondrial (**d**) and the cytosol (**e**) fractions of Jurkat cells which were co-incubated with TEX1 for 3, 6 or 16 h. Mitochondrial and cytosol fractions of Jurkat cells were prepared as described in Methods. β-Actin and Cox IV served as controls for equal loading of the cytosol and mitochondrial fractions, respectively. **f**, **g** Western blots of proteins in the mitochondrial (**f**) and the cytosol (**g**) fractions of Jurkat cells which were co-incubated with TEX2 or HaCaT sEV for 16 or 24 h. AIF apoptosis-inducing factor. **h**, **i** Effects of TEX1 and TEX2 on expression levels of the apoptosis related proteins in CD8+ Jurkat T cells after 6 h co-incubation. Representative histograms are shown in (**i**) and quantitative mean fluorescence intensity (MFI) values are shown in (**h**). Data are means ± SD from 3 independent experiments.

The ability of TEX interacting with T cells to induce apoptosis regardless of various blocks imposed to prevent it suggested that TEX introduced mechanisms able to drive apoptosis in recipient cells. Specifically, TEX could deliver caspases, promote activation of caspases, or suppress expression/activity of survival proteins in the recipient T cells. When, however, recipient Jurkat T cells were pre-treated with a caspase 8 inhibitor (Z-IETD-FMK) or with a pan-caspase inhibitor (Z-VAD-FMK), TEX entry was not impeded, and apoptosis, while significantly reduced (~30%), was not eliminated (Fig. 7a, b**)**. T cell apoptosis was not reduced further when both inhibitors were combined. TEX-mediated apoptosis of activated CD4^+^ T cells pretreated with either caspase inhibitor was not inhibited at all (Fig. 7c). Further, when we pre-treated Jurkat T cells with necrostatin, an inhibitor of necrosis, cell death induced by TEX was not blocked.

### TEX induce intrinsic apoptosis in recipient T cells

To further study the mechanisms underlying TEX induced T cell apoptosis, CD8^+^Jurkat cells co-incubated with TEX for 3–16 h were permeabilized, lysed and separated into the mitochondrial and the cytosolic fractions as described in Methods. Figure 7d shows that in the mitochondria, cytochrome C, Smac, BcL-2, and Bcl-xL levels decreased at 6 h and 16 h of coincubation with TEX1 relative to PBS controls. In the cytosol (Fig. 7e), cytochrome C and Smac were detectable at 3 h and their levels increased at 16 h. Further, cleavage of caspase-3 (but not of caspase-8) and PARP were seen in the cytosol within 3 h of co-incubation of Jurkat T cells with TEX1 and increased with the co-incubation time. Bcl-xL levels also increased with time, and cFLIP cleavage was mildly enhanced. These data show that TEX1 taken up by activated T cells induced cytochrome C, Smac, Bcl-2, BcL-xL release from mitochondria and thereby unleashed intrinsic apoptosis leading to T cell death. The levels of these proteins increase in the cytosol, although not for BcL-2, which is rapidly degraded. We also co-incubated CD8+ Jurkat cells with TEX2 and observed only weak cytochrome C and Smad release from mitochondria after 24 h. HaCaT sEV had no discernible effects on cytochrome C or Smac release from mitochondria even after 24 h co-incubation (Fig. 7f). Nevertheless, the cytosol fractions of Jurkat co-incubated with TEX 2 showed the presence of cytochrome C, Smac and caspase-8 fragments at 16 and 24 h but no evidence for caspase-3 or PARP cleavage (Fig. 7g). Only low levels of cytochrome C and Smac were seen in cytosol of Jurkat co-incubated with HaCaT sEv for 24 h. Changes in BcL-2 and BcL-xL levels were not apparent in mitochondria at 24 h, although accumulations of BcL-xL in the cytosol were seen at 16 and 24 h and were greater in TEX2 than in HaCaT. In aggregate, these data indicate that the rate and magnitude of intrinsic apoptosis are different for TEX1, TEX2 and HaCaT: while TEX1 effectively induced intrinsic apoptosis in T cells already at 6 h, TEX2 used at the same protein concentration required at least 16 h. HaCaT sEV had minimal effects on cytochrome C release at 24 h only. Apoptosis inducing factor (AIF), which initiates a caspase-independent apoptosis by DNA fragmentation, was included in the experiment (Fig. 7g, f) to show that TEX2 induced its release from mitochondria.

We previously reported that EVs from cancer plasma promoted apoptosis by downregulating expression of survival proteins in recipient T cells^27^. The data in Fig. 7h, i show that when Jurkat Tells were co-incubated with TEX1 or TEX2 at the concentration that induced ~40% apoptosis, expression levels of Bcl-2, BcL-xL and cFLIP, as determined by flow cytometry of intact cells, were altered relative to PBS controls. Importantly, Bax levels were decreased in T cells. Bax is known to translocate from the cytosol to mitochondria during intrinsic apoptosis and induce cytochrome C release. The observed reduction in levels of Bax in T cells treated with TEX1 or TEX 2 confirms the WB data showing cytochrome C and Smac accumulations in the cytosol (Fig. 7e, g**)**.

## Discussion

While immunotherapy (IT) of cancer has been a success, with objective response rates reaching ~50% in some solid malignancies, many patients fail to respond to this form of therapy for reasons that are not clear^28^. Tumor-induced immune suppression, a well-known hallmark of cancer, might be responsible for patients’ unresponsiveness to IT. Among a wide range of cancer-derived negative regulators known to contribute to dysfunction or exhaustion of effector T cells, tumor-derived sEV aka TEX are emerging as major negative regulators of anti-tumor immunity and of response to IT. Our previous reports showed that sEV isolated from plasma of patients with melanoma or HNSCC suppressed functions (activation, proliferation, cytokine production) of activated primary human T cells and induced T cell apoptosis^16,27,29^. We suggested that simultaneous delivery by TEX of multiple various signals to T cells leads to suppression of T cell functions and may culminate in cell death^16^. Here, we showed that sEV isolated from plasma of TNBC patients or from supernatants of TNBC CLs selectively induced rapid concentration dependent apoptosis of activated CD4^+^ and CD8^+^T cells, while activated primary B or NK cells were relatively resistant to TEX-induced apoptosis, despite the presence of Fas on the cell surface (Fig. 4a). Differential expression of receptor proteins on these immune cells might in part explain their relative resistance to TEX-mediated apoptosis, although it may be the sum of stimulatory vs suppressive surface protein expression that is more relevant than expression levels of individual proteins. We speculate that the TEX entry into B or NK cells might not induce apoptosis, because it activates alternate molecular pathways in these cells. Specifically, our earlier data suggest that NK cells downregulate the NKG2D expression and cytolytic functions after co-incubation with TEX^30^, while B cells tend to activate the adenosinergic pathways, leading to adenosine production and inhibition of T cell proliferation^31^.

Uptake of TEX by T cells was relatively slow as we have previously shown^26^, and a 10–15 min delay in TEX uptake might be sufficient for the initiation of apoptotic signals via the receptor-ligand interactions.^26,29^ Surprisingly, however, blocking of TEX-induced signaling with Abs neutralizing death receptor/ligands on the sEV surface failed to reduce T cell apoptosis, although the TEX entry into recipient T cells was unimpaired. Using pre-titered neutralizing mAbs specific for death/receptor ligands singly or in combination to block TEX-induced signaling only minimally reduced apoptosis (~10% of control). The failure to block T cell apoptosis by disrupting signaling of the death/receptor ligands with proteinase K or heat treatments, which only partially blocked but did not eliminate apoptosis, suggests that TEX might use other mechanisms to regulate T cell death. Reasoning that apoptosis of recipient T cells is a consequence of sEV uptake, we attempted to interfere with the vesicle internalization by T cells, B cells and NK cells. Neither sEV pre-treatment with proteinase K nor protein denaturation by heat prevented sEV uptake into various immune cells. Also, sEV uptake by immune cells was only reduced but not prevented by pre-treatments with Dynasore, Cytochalasin D or PIT Stop 2, and the recipient B or NK cells resisted apoptosis. Further, inhibition of pan-caspase or caspase 8 activity in T cells co-incubated with TEX also failed to stop apoptosis. Together, these results suggest that once TEX enter the recipient T cells, apoptosis becomes unavoidable and that it is independent of surface signaling by death receptor/ligands.

One potential explanation for the failure to arrest the relentless apoptotic pathway induced by TEX with Abs blocking death receptor/ligand signaling might be due to physical stress imposed by vesicles in recipient T cells. The entry of sEV into any cell results in the delivery of various extraneous materials carried by vesicles to the recipient cell cytosol. Conceivably, this leads to cellular stress and molecular alterations known as stress associated molecular patterns (SAMPs) that could explain relentless apoptosis we observed after sEV entered T cells^21,22^. Cell stress-induced stimuli are known to upregulate and activate death receptors, promoting apoptosis^21,22^. We showed that cell signals inducing cell stress, including co-incubation of T cells with TEX, provoked intrinsic apoptosis. Activated T cell were more sensitive to this TEX-mediated apoptosis than other immune cells. This does not imply that TEX ability to induce intrinsic apoptosis is T cell specific. Rather, tumor-derived contents of TEX, together with prolonged kinetics of TEX entry into T cells (Fig. 3), culminate in rapid T cell death resulting from mitochondrial damage by an unknown TEX-associated factor(s). The mechanisms of cell stress are multiple and are currently under intense scrutiny^32^. The major stress response pathways characterized to date include: the ER-associated unfolded protein response (UPR), the DNA damage response (DDR), the mitochondrial UPR and the heat shock response (HSR). These stress-induced alterations can be induced by genetic, metabolic, or environmental factors^32^. TEX, which recapitulate the content of tumor cells, carry on their surface or in their lumen a broad variety of proteins, lipids, glycans and nucleic acids that upon entry create stress in a variety of recipient cells. As mitochondria are highly sensitive to cellular stress, we hypothesized that TEX entering into a T cell alter mitochondrial integrity and functions. As expected, changes in pro-apoptotic and anti-apoptotic protein levels were observed the mitochondria and cytosol of Jurkat cells following their co-incubation with TEX1 or TEX2 for various time periods (Fig. 7d-g). These changes, especially cytochrome C and Smac as well BcL-2 and BcL-xL release from mitochondria were consistent with TEX-driven intrinsic apoptosis that was already evident at 3 h and progressively enhanced at 6 h and 16 h of co-incubation. sEV produced by non-malignant cells (e.g., HaCaT) induced only minimal apoptosis after 24 h of co-incubation with activated T cells. Accumulations of cleaved fragments of caspase 3, PARP and cFLIP in cytosol of Jurkat cells co-incubated with TEX1 confirmed that intrinsic apoptosis is one of the mechanisms underlying the demise of activated T cells interacting with TEX. We also observed that flow cytometry of T cells co-incubated with TEX showed downregulated expression levels of the survival proteins and Bax in CD8^+^T cells co-incubated with TEX, consistent with ongoing intrinsic apoptosis. This appears to be the mechanism generally utilized by TEX produced by different types of tumor cells: we obtained the same results with TEX obtained from cell lines and plasma of patients with melanoma or HNSCC.

Negative signaling of TEX in TNBC, and other solid tumors contributes to resistance of tumor cells to oncological immune therapies (ITs). Adoptively delivered activated immune cells, T cells, NK cells or engineered CAR T cells, are in special danger from TEX which are present in large numbers in the circulation of patients with advanced cancer^16,33^. We have previously reported on the therapeutic failure of adoptively transferred activated NK cells to patients with acute myelogenous leukemia (AML) that was attributable to the presence of highly immunosuppressive TEX in the patients’ circulation^34^. Taken together, our studies suggest that effector T cells are simultaneously targeted by multiple inhibitory signals TEX deliver, including cellular stress signals that lead to mitochondrial dysfunction and relentless intrinsic T cell apoptosis.

The current anti-cancer immune therapies are based on the notion that a recovery of T cells from tumor-induced suppression is necessary for the favorable therapeutic response. However, the abundance of immunosuppressive TEX in body fluids of patients with advanced cancers counterbalances restorative effects of IT. Disarming of TEX with mAbs or inhibitors targeting specific receptor-ligand signaling and blocking external T cell apoptosis is unlikely to work, as the entry of sEV into T cells leads to intrinsic apoptosis that is largely independent of death receptor/ligand signaling. Strategies of reducing TEX-induced cellular stress to rescue activated T cells might be considered such as removal of the sEV excess from cancer patients’ circulation by affinity plasmapheresis^35^ or suppression of TEX release with calcium channel blockers^36^. Studies are urgently needed to protect activated T cells from relentless TEX and to prevent TEX from enhancing tumor resistance to onco-immune therapies.

## Methods

### Cell lines

Human metastatic Triple Negative Breast Cancer Cell Lines (TNBC-CL) MDA-MB-231 (CL 1) and MDA-MB-436 (CL 2) as well as a non-malignant cell line, HaCaT (immortalized human keratinocytes), were obtained from ATCC including the authentication certificate (genomic profiling) and were cultured in Dulbecco’s modified Eagle’s medium (Gibco Fisher Scientific). All cell lines were tested for Mycoplasma using Lonza’s MycoAlert detection assay. For TEX production, 4 × 10^6^ cells were cultured in 25 mL medium in a 150 cm^2^ flask for 72 h. The supernatant was collected for sEV isolation as previously described^37^. Pre-clarified supernatant was concentrated to 1 mL using a Vivaspin-20, 100,000 MWCO (Sartorius Corp, Bohemia, NY, USA) and placed on a Sepharose 2B column for sEV isolation by size exclusion chromatography (SEC)^38^. The Jurkat cell line expressing surface CD8 protein was obtained from Dr H. Rabinowich (Department of Pathology, University of Pittsburgh, PA) and cultured in RPMI medium^39^. All culture media were supplemented with 10% (v/v) exosome-depleted and heat-inactivated fetal bovine serum (FBS, Gibco), 100 U/mL penicillin, and 100 µg/mL streptomycin. Cells were maintained at 37 °C and in an atmosphere of 5% CO_2_ in air.

### Plasma samples

Plasma specimens from TNBC patients (TNBC-Pts) were collected at the Magee-Women’s Hospital under the IRB approved protocol # 04–162 and were obtained from the University of Pittsburgh Biospecimen Core (http://www.pittbiospecimencore.pitt.edu). All specimens are annotated. For this study, banked plasma specimens collected at the time of diagnosis were obtained from five randomly selected female patients (aged 25–45 yrs) with metastatic TNBC. Plasma was aliquoted in 1 mL vials and stored in liquid nitrogen until it was thawed for sEV isolation. Plasma samples of age- and sex-matched healthy donors (HD’s) were collected from volunteers who signed a consent form approved by the IRB (# 04–162).

### sEV isolation and characterization

Thawed plasma pre-cleared by sequential centrifugations and ultrafiltration and was used for sEV isolation by SEC, eluting vesicles with PBS^38^. The bulk of sEV eluted in fraction #4^38^. Isolated sEV were concentrated using 100,000 MWCO Vivaspin 500 centrifugal concentrators (Sartorius Corp.). sEV protein concentrations were measured using a BCA protein assay kit (Pierce Biotechnology, Rockford, IL). Transmission electron microscopy (TEM) of vesicles in the SEC Fraction #4 was performed at the Center for Biologic Imaging at the University of Pittsburgh^38^. The vesicles on copper grids stained with 1% (v/v) uranyl acetate in ddH_2_O were visualized using TEM (model JEOL JEM-1011). The concentration and size distribution of sEV or TEX were measured by nanoparticle tracking analysis (NTA) using NanoSight 300 (Malvern, UK). The vesicles were diluted in ddH_2_O, and then the video image was captured at the camera level of 14. The captured videos were analyzed using NTA software, maintaining the screen gain and the detection threshold at 1 and 5, respectively. To determine mean particle size/concentration in each sample, five consecutive measurements were obtained and averaged. Western blots were used to evaluate sEV or TEX protein profiles. Vesicle aliquots (10 µg protein) were lysed with Laemmli sample buffer (Bio-Rad Laboratories, Hercules, CA, USA), separated using 4–15% SDS/PAGE gels, and after transfer from gels to the polyvinylidene fluoride (PVDF) membranes, proteins were detected using Abs specific for antigens carried by sEV (see Supplementary Table 3)^38^.

### Isolation of primary human immune cells

Peripheral blood mononuclear cells (PBMCs) were isolated from peripheral blood of HDs by centrifugation on Ficoll-Paque Plus (GE Healthcare Lifesciences) gradients^38^. CD4^+^ T, CD8^+^ T, NK and B cells were isolated from PBMCs using negative selection-based cell isolation kits from Miltenyi, #130-096-495, #136-096-533, Stem Cell Technologies, #19055, and Biolegend #480061, respectively, following manufacturers’ protocol. Isolated cells were cultured in RPMI medium supplemented with 10% (v/v) exosome-depleted and heat-inactivated FBS at 37 °C in the atmosphere of 5% CO_2_ in air^16^. T cells were activated with CD3/CD28 T cell activator (25 µl/mL, Stem Cell) and IL-2 (150 IU/mL, Peprotech) at 1 × 10^6^ cells/mL in RPMI medium for 12 h. NK cells were activated by 48 h incubation with IL-2 (100 IU/mL, Peprotech) and IL-15 (150 IU/mL, Peprotech) at 1 × 10^6^ cells/mL. B cells were activated by 12 h incubation with IL-4 (100 IU/mL, R & D System) and CD40L (0.5 ug/mL, Cell Signaling Technology, #3583 S) at 1 × 10^6^ cells/mL.

### Uptake of labeled TEX by activated primary human immune cells

sEV isolated from supernatants of cultured MDA-MB-231 (TEX1) and MDA-MB-436 (TEX2) cells were labeled with the PKH26 red fluorescent dye (Sigma-Aldrich, St. Louis, MO)^30^. Briefly, a TEX aliquot (30 µg protein/500 µL PBS) was mixed with 500 µL Diluent C containing 2 µL of the PKH26 dye and incubated for 5 min at RT in the dark. The reaction was quenched by adding 1 mL of 1% BSA to the reaction mixture. The PKH26-labeled TEX were washed 5x with 1 mL PBS using an Amicon Ultra filter (100,000 MWCO) to remove the PKH26 dye excess and were resuspended in 100 µL of PBS. For cellular uptake, freshly labeled TEX (10 µL) were co-incubated with 1 × 10^5^ CD8^+^ Jurkat cells in 100 µL RPMI medium for 0.25, 0.5, 1, 2 and 6 h at 37 °C. The dye alone control (no TEX) was included in all uptake experiments and was consistently negative. To compare TEX 1 uptake by activated primary CD4^+^T and CD8^+^T cells, PKH26-labeled vesicles (10 µL) were co-incubated with 1 × 10^5^ CD4^+^T or CD8^+^T cells in 100 µL RPMI medium for 0.5, 1 and 2 h at 37 °C. Following incubation, cells were washed with PBS (×3) and TEX uptake was analyzed by flow cytometry using a Cytoflex (Beckman Coulter). Uptake of vesicles by CD8^+^ Jurkat cells was also imaged in a Zeiss fluorescent microscope at 40x mag.

### Inhibition of TEX uptake by immune cells

TEX were pretreated with proteinase K (PK) or subjected to heat to block their uptake by Jurkat T cells. The PKH26-labeled TEX (10 µL) were treated with 1 µg PK (New England Biolabs, #P8107S) for 30 min at 37 °C and were used for analysis immediately after quenching with 1 µL of 20X PMSF. For heat-inactivation, TEX were first incubated at 80 °C for 1 h and then labeled with the PKH26 dye. Freshly labeled TEX were used in co-incubation experiments with CD8^+^ Jurkat T cells (5 ×10^5^ in 100 µL RPMI medium) to determine the time course of TEX uptake. The PKH26 dye alone (no TEX) controls were included in all co-incubation experiments. Images of TEX uptake by Jurkat T cells were acquired at 0, 0.5, 2 and 6 h in a Zeiss fluorescent microscope at 40x mag.

To evaluate and compare the effects of heat inactivation or PK treatment on TEX uptake by Jurkat T cells, the PKH26-labeled vesicles pretreated with heat or PK as described above were co-incubated with CD8^+^Jurkat cells (5 ×10^5^ in 100 µL RPMI medium) for 2 h to allow for the vesicle entry. Following incubation, Jurkat T cells were washed with PBS (x3) and fixed by incubating with 2% paraformaldehyde for 10 min at RT. Fixed cells were washed with PBS x3, permeabilized, and blocked by incubating with 2% BSA containing 0.05% Triton-X for 30 min. The permeabilized cells were incubated with AF488-conjugated phalloidin antibody (1:50, Thermo Fisher #A12379) in 0.5% BSA with 0.05% Triton-X for 1 h. Cells were washed with 0.05% Triton-X (x3) and finally dispersed into 200 µL PBS. Cells were cytocentrifuged onto glass slides and cover slipped using DAPI mounting medium (DAPI fluoro-mount G, Southern Biotech #0100-20). Cells were imaged at 40x mag using an Olympus Fluoview 1000 confocal microscope.

To block TEX entry into T cells, CD8^+^ Jurkat cells (5 ×10^5^ in 100 µL RPMI medium) were pretreated with the following blocking agents for 30 min: Cytochalasin-D, Dynosore, Pit Stop 2 or anti-Fas Ab (see Table 1). The optimal concentrations of all blocking reagents were determined in preliminary titration experiments. Following 30 min blocking, T cells were co-incubated with PKH26 labeled TEX (10 µL) for 2 h, washed, and processed for imaging as described above. Cells were imaged at 40x mag under an Olympus Fluoview 1000 confocal microscope.

### On-bead flow cytometry for immunoregulatory proteins on the vesicle surface

On-bead flow cytometry of sEV or TEX was performed as previously described^40^. Briefly, sEV or TEX (10 µg/100 µL PBS) were incubated with a cocktail of biotin-labeled anti-CD63 mAb (0.5 µg, Biolegend, #353018) and biotin-labeled anti-CD9 mAb (0.5 µg, Biolegend, #349514) for 2 h at RT. Next, streptavidin coated magnetic beads (50 µL aliquot; ExoCap™, MBL International, Woburn, MA) were added to the vesicle-Ab mixtures and incubated for 2 h at RT. The bead/Ab/vesicle complexes were washed with PBS and dispersed in 50 µL PBS. For detection of target antigens, 4 µL aliquots of the complex were dispersed in 100 µL PBS and blocked with 2% mouse serum. The pre-titered labeled detection Abs (see Table 1) were added; beads were washed with PBS and diluted in PBS for antigen detection using flow cytometry. Antigen expression levels were measured as relative fluorescence intensity (RFI) calculated as the ratio of Ag RFI/isotype control RFI. The immunosuppressive RFI score was calculated for each vesicle lot (cell line or patient derived) by summarizing the RFI values of proteins mediating suppression (see Supplementary Table 1A). Similarly, the immunostimulatory RFI score was calculated for each vesicle lot by summarizing the RFI values for immunostimulatory proteins (see Supplementary Table 1B). For each vesicle lot, the Stim/Supp Ratio was determined as stimulatory RFI score/suppressive RFI score.

### Apoptosis of immune cells co-incubated with TEX

Activated primary human CD4^+^Tcells, CD8^+^T cells, B cells, NK cells or cultured CD8^+^ Jurkat T cells were plated in wells of a U-bottom 96-well plate at the concentration of 1 × 10^5^ cells/well in RPMI medium. Freshly prepared sEV or TEX (2.5 µg, 5 µg or 10 µg protein) were added to the cells in 100 µL RPMI medium and co-incubated for 6 h. As controls for TEX, sEV isolated from supernatant of a non- malignant cell line (HaCat) or sEV isolated from plasma of HDs were used. The target cells were also incubated with an equal volume of PBS alone. Following incubation, apoptosis was measured by flow cytometry using FITC Annexin-V (ANXV) Apoptosis Detection Kit (BD Biosciences, #55647) in a Cytoflex flow cytometer (Beckman). Gating strategy is illustrated in Supplementary Fig. 6. In preliminary experiments, flow cytometry based TUNEL assay (Elabscience) was used as recommended by the manufacturer to confirm DNA fragmentation in target cells co-incubated with TEX.

### Apoptosis blocking experiments

For blocking experiments, two strategies were investigated: (a) activated T cells were plated in wells of a 96-well plate (1 × 10^5^ cells/well) and were pre-incubated with blocking Abs or inhibitors for 30 min before the addition of TEX (2.5µg-5µg protein) and subsequent 6 h co-incubation or (b) blocking Abs/inhibitors were placed in wells prior to the simultaneous addition of vesicles and CD8^+^Jurkat T cells and remained in co-cultures for 6 h preceding Annexin-binding assays. These blocking strategies yielded similar results, and the first strategy was used in the reported experiments. Responder activated T cells were treated with the neutralizing Abs/specific inhibitors (see Table 1) or the relevant IgG controls. Following the co-incubation of T cells with inhibitors +/− TEX or +/− sEV, apoptosis of CD8^+^ T cells was measured by flow cytometry using a FITC Annexin-V Apoptosis KitT. The combined percent values for early apoptosis (ANXV^+^ PI^˗^) and late apoptosis (ANXV^+^ PI^+^) were used for comparisons between groups.

### Subcellular fractionation of Jurkat T cells co-incubated with TEX

CD8^+^Jurkat cells (2 ×10^6^/well) plated in wells of 6-well plates were co-incubated with TEX (10 µg/mL)isolated from supernatants of MDA-MB231 and MDA-MB436 or with PBS as control for 3, 6 16 or 24 h as described above. The cells were harvested and sub-fractionated as previously reported into the mitochondrial and cytosol fractions^27^. Briefly, the cells were suspended in digitonin permeabilization buffer (50 mmol/l sucrose, 137 mmol/l NaCl, 70 mmol/l KCl, 4.3 mmol/l Na2HPO4, 1.4 mmol/l K2HPO4, 0.2 mg/ml digitonin and 0.1% Hydorol M), vortexed and incubated on ice for 5 min. The cells were then centrifuged at 1000 × g for 5 min at 4 °C. The supernatant contained the cytosolic fraction. The remaining pellet was resuspended in RIPA buffer (50 mmol/l Tris-HCl (pH 7.4), 150 mmol/l NaCl, 1 mmol/l EDTA, 1 mmol/l EGTA, 1% Triton X-100, 1% sodium deoxycholate and 0.1% SDS), vortexed and incubated on ice for 5 min. The lysate was vortexed and centrifuged at 10 000 × g for 10 min at 4 °C. The supernatant contained the mitochondrial fraction. The subcellular fractions were resolved by SDS-PAGE, and the selected pro- or anti-apoptotic proteins were detected by immunoblotting. β-Actin and Cox IV served as controls for equal loading for the cytosol and mitochondrial fractions, respectively. The following antibodies used for WBs were purchased from Santa Cruz: Beta-actin Ab sc-47778; Bcl-2 Ab sc-509; Bcl-XL Ab sc-634; Cyto C Ab sc-131356; Caspase-3 Ab sc-7148; FLIP Ab sc-5276; Smac Ab sc-22766. Abs to Caspase-8, 551242 and Abs to PARP, 556362 were purchased from BD Pharmingen. Abs to COX IV,4844 were purchased from Cell Signaling.

### Assessment of survival proteins in T cells co-incubated with TEX

Expression of anti-apoptotic proteins (BCL-2, BCL-XL and cFLIP) and the pro-apoptotic protein BAX was evaluated in CD8^+^ Jurkat T cells. TEX (5 µg protein) were co-incubated with CD8+ Jurkat T cells (5 ×10^5^ in 100 µL RPMI medium) for 6 h. Cells were washed with PBS, fixed with 1% paraformaldehyde in PBS at RT for 10 min and permeabilized with 0.1% saponin in PBS for 15 min at 4 °C. Next, cells were stained with PE- or AF488-conjugated Abs specific for human BCL-2, BCL-XL, BAX or unconjugated anti-cFLIP Abs (Supplementary Table 2). Cells were washed x3 with 0.1% saponin. Cells stained with unconjugated Abs were further stained with PE-conjugated goat-anti mouse IgG for 30 min at 4 °C. Finally, cells were washed with 0.1% saponin and evaluated for the content of pro- and anti-apoptotic proteins using flow cytometry (Cytoflex).

### Statistics and reproducibility

Results are presented as means ± SD. Experiments were performed in triplicate. Statistical analyses were performed using graph pad prism 8.3. software (GraphPad Software, Inc., La Jolla, CA 92037 USA). The differences between groups were measured by t-test and one-way ANOVA followed by Dunnett’s post hoc test or by Mann-Whitney tests. *P-*values < 0.05 were considered as significant.

### Reporting summary

Further information on research design is available in the Nature Portfolio Reporting Summary linked to this article.

### Supplementary information

Supplementary Information

Description of Additional Supplementary Files

Supplementary Data 1

Reporting Summary

## Data Availability

All data supporting the findings of this study are available within the paper, in Supplementary Data 1 and in the Supplementary Information file, including the source data as Original Western Blots in Supplementary Fig. 7. All data are available from the senior or corresponding author upon a reasonable request.
