# Supplementary material for: Small EV in plasma of triple negative breast cancer patients induce intrinsic apoptosis in activated T cells
Source: Commun Biol. 2023 Aug 4;6:815. doi: 10.1038/s42003-023-05169-3 (PMC10403597; doi:10.1038/s42003-023-05169-3)

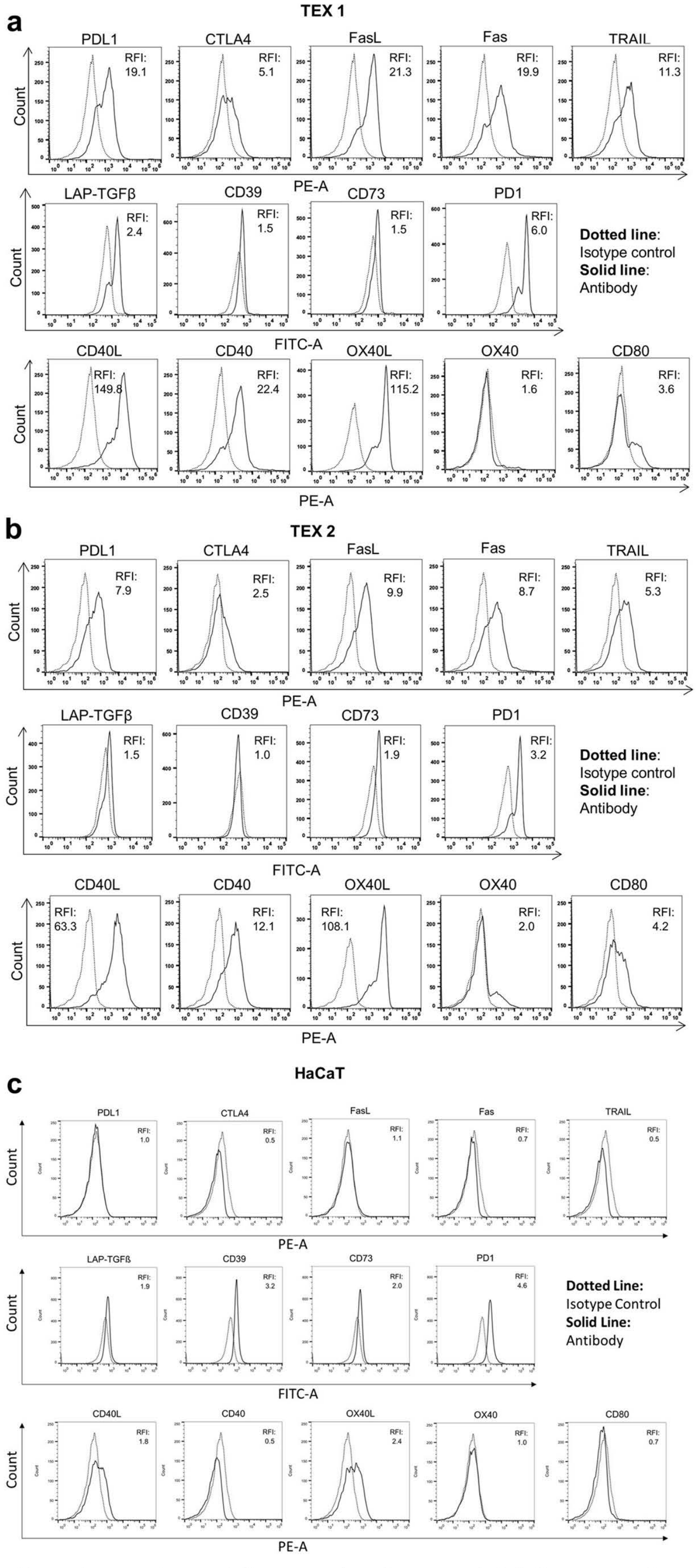

Supplementary Figure 1. Immunoinhibitory and immunostimulatory protein expression levels in TEX1 (a) in TEX2 (b) and in HaCat sEV (c). On-bead flow cytometry shows relative fluorescence intensity (RFI) values.

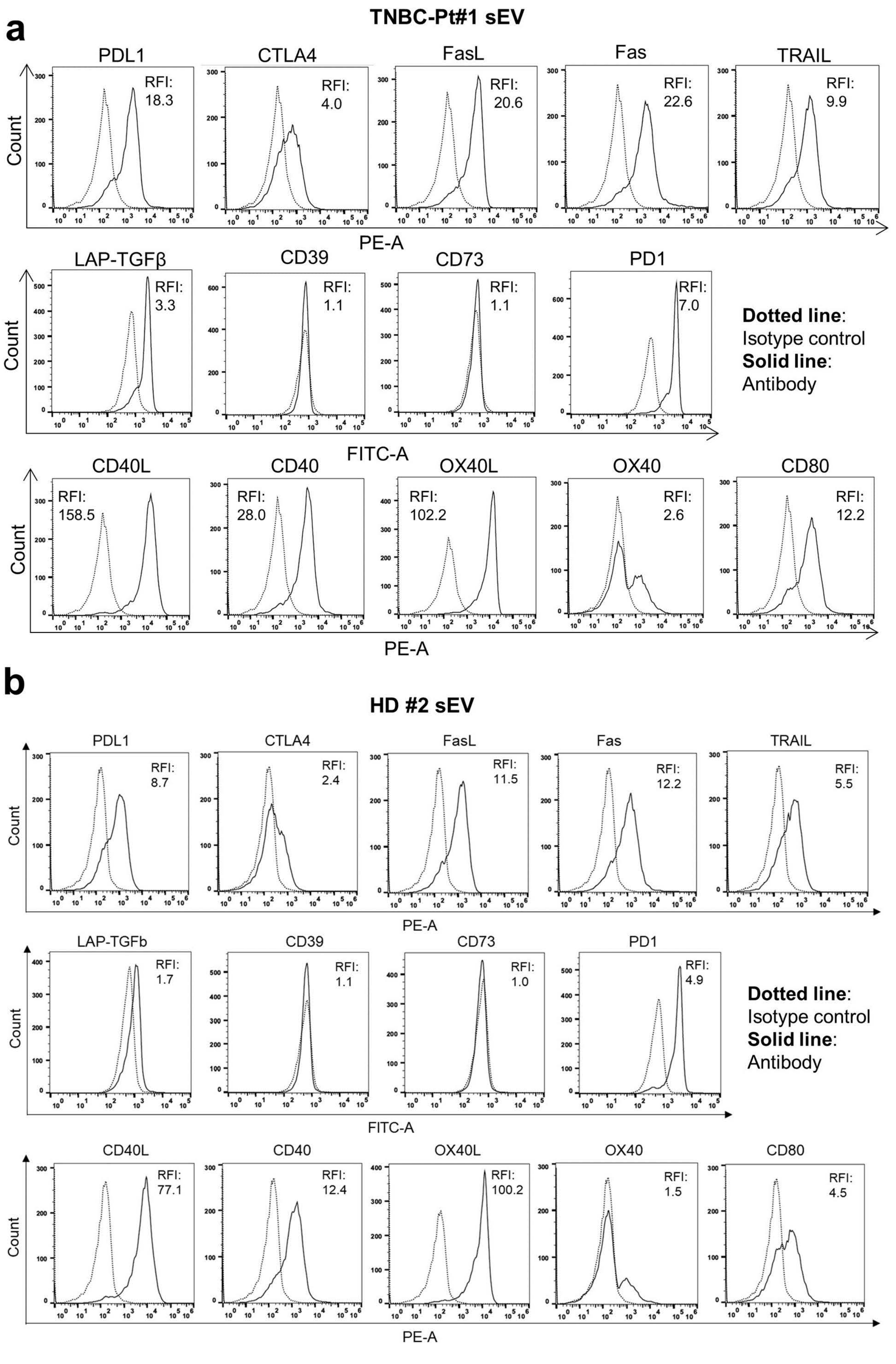

Supplementary Figure 2. Immunoinhibitory and immunostimulatory protein expression levels in plasma sEV from TNBC Pt #1 (a) and in plasma sEV from HD #2 (b). On-bead flow cytometry shows relative fluorescence intensity (RFI) values.

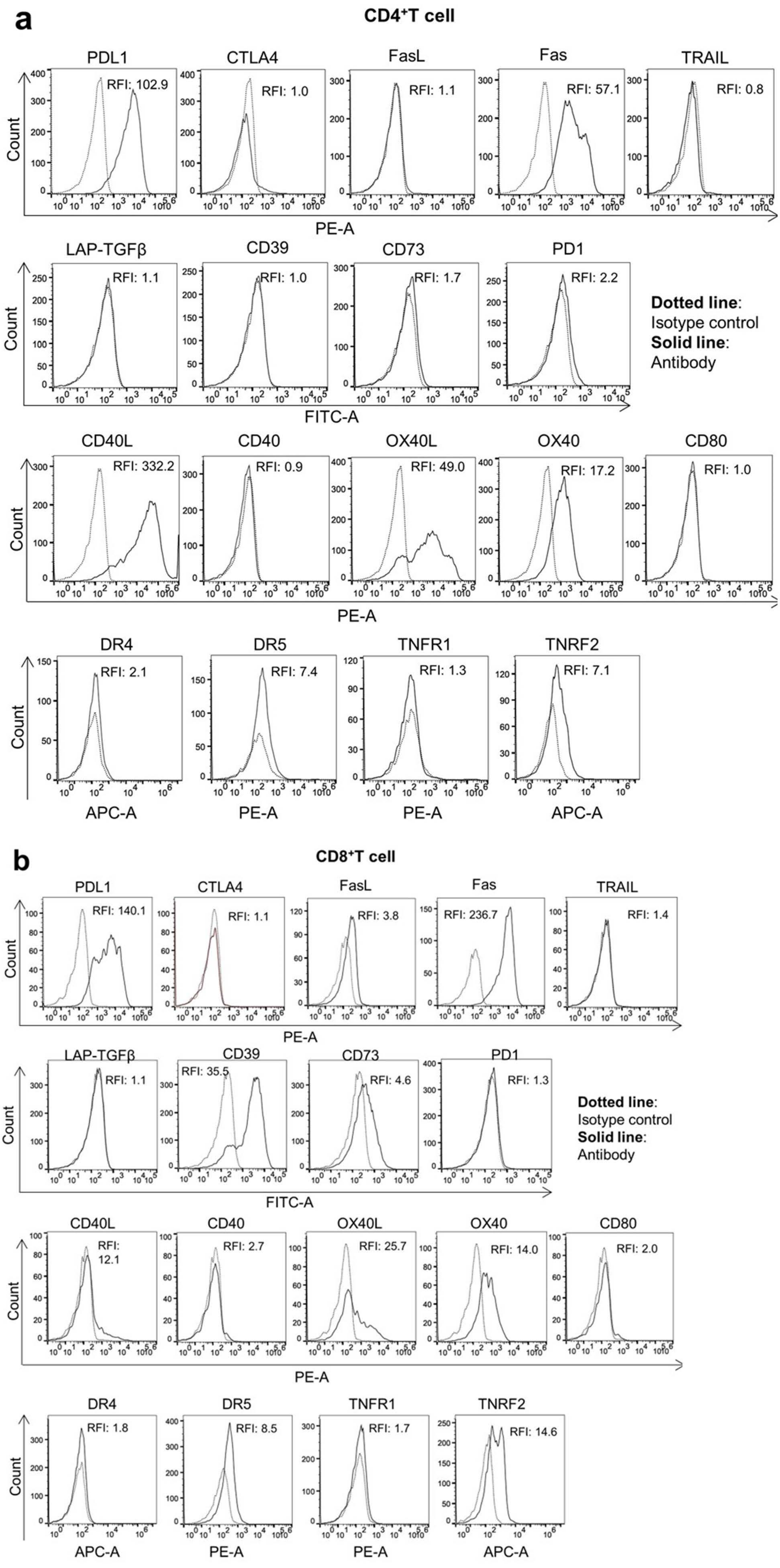

Supplementary Figure 3. Immunoinhibitory and immunostimulatory protein expression levels on the surface of activated CD4+T cells (a) and on the surface of activated CD8+T cells (b). Flow cytometry shows RFI values for various surface markers.

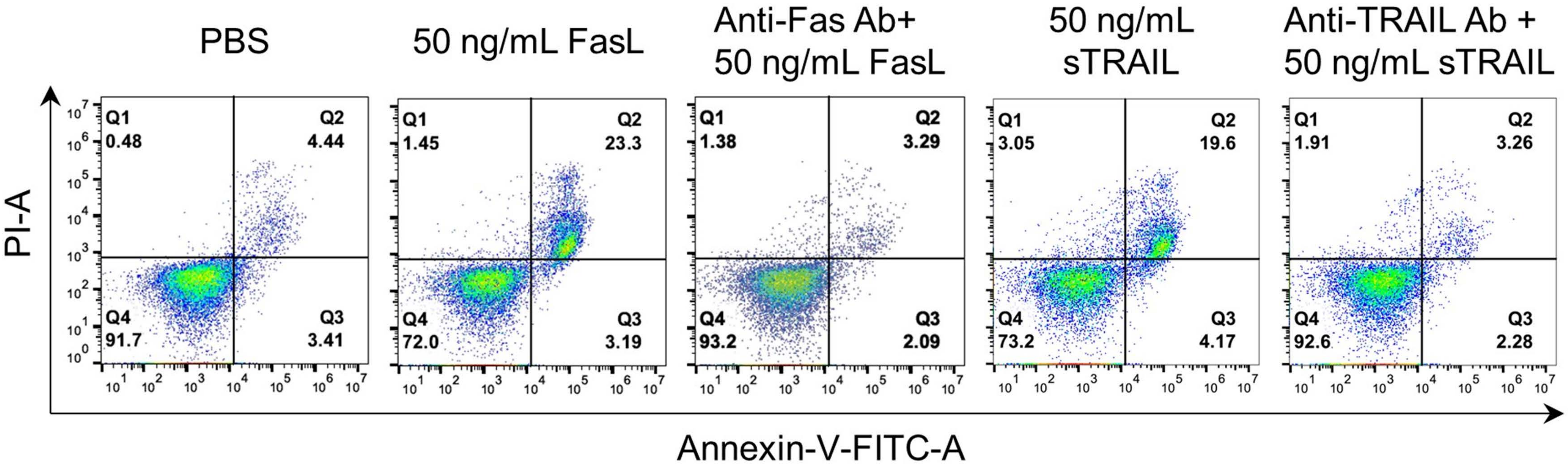

Supplementary Figure 4. Validation of neutralizing activity of anti-Fas Abs and anti-TRAIL Abs used in blocking experiments. Representative data for apoptosis induced by FasL (Peprotech, #31-03H) or soluble TRAIL (Peprotech, # 310-04) in CD8+ Jurkat cells following 6h incubation. For blocking experiments, cells were pre-incubated with anti-Fas Ab (10μ/mL, Millipore, #05-338) or anti-TRAIL Ab (10μg/mL, Thermo Fisher, #16-4714-82) for 30 min before co-incubation for 6h with FasL or TRAIL, respectively.

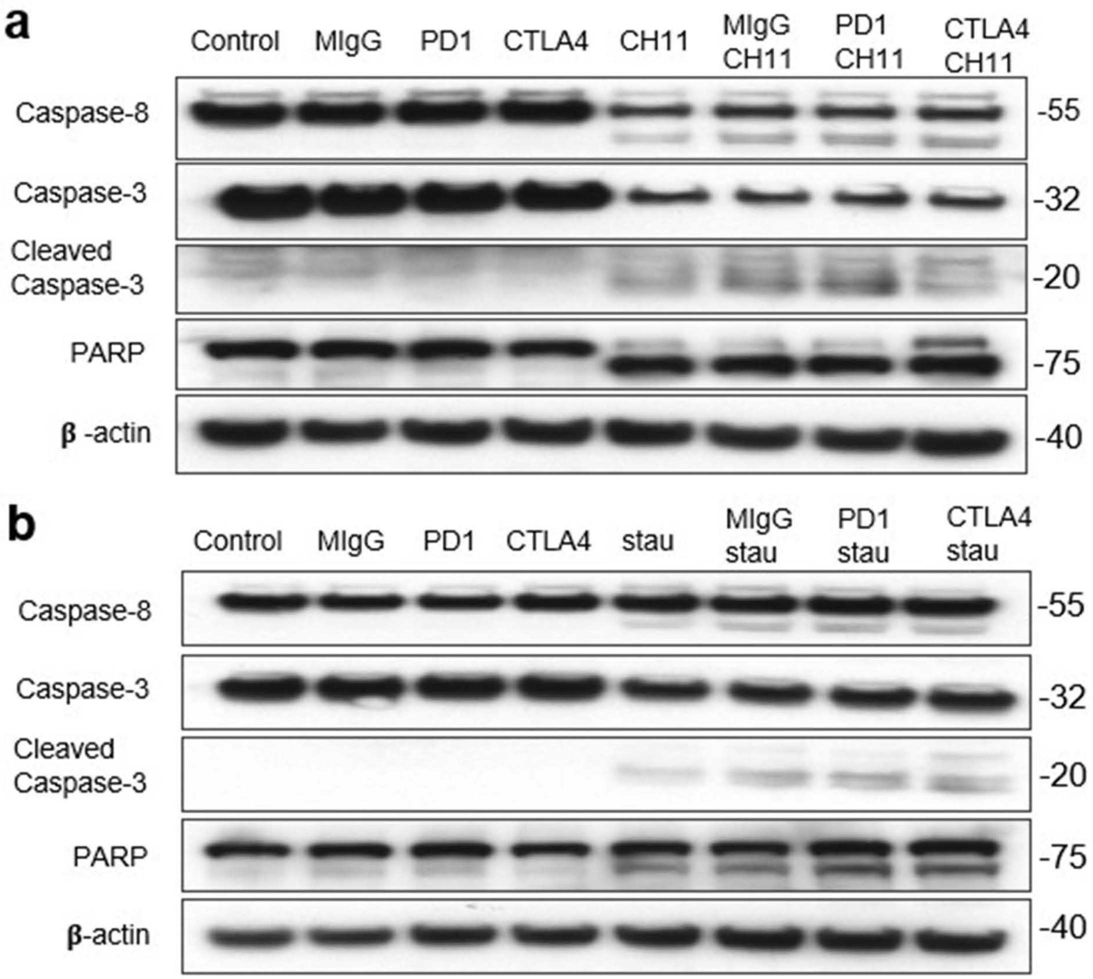

Supplementary Figure 5. Apoptosis of Jurkat CD8+ T cells induced by anti-Fas (CH11) Ab (a) or by staurosporine (b) is not blocked by neutralizing antibodies specific for PD-1 or CTLA4. Jurkat T cells were incubated with neutralizing anti-PD1 Abs (10μg/mL, Abcam ab52587 M), anti-CTLA4 Abs (20μg/mL, Santa Cruz, sc-376016 M) or control mouse IgG (10μg/mL) for 30 min. Next, apoptosis was induced by anti-Fas Ab (CH11; 10ng/ml, Upstate 05-201 M) or staurosporine (0.5uM, Sigma S6942), After 16 h incubation, lysates of Jurkat cells were prepared using a cocktail containing 1% Nonidet P-40, 20 mM Tris-HCl, pH 7.4, 137 mM NaCl, 10% glycerol, 1 mM PMSF ,10 μg/ml leupeptin, and 10 μg/ml aprotinin. After proteins in the cell lysate wwere resolved by SDS-PAGE, the selected pro-apoptotic proteins were detected by immunoblots using the following Abs: anti-caspase-8 Ab, BD Pharmingen 551242 M; anti-PARP Ab, BD Pharmingen 556362 M; anti-caspase-3 Ab, Santa Cruz sc-7148 R; and anti-β-actin Ab, Santa Cruz 47778 M. The immunoblots shown in (a) and (b) indicate that neutralizing anti-PD1 Abs or CTLA4 Abs did not block cleavage of caspase-3, caspase-8 or PARP in Jurkat T cells undergoing extrinsic apoptosis induced by agonistic CH-11 Ab or staurosporine.

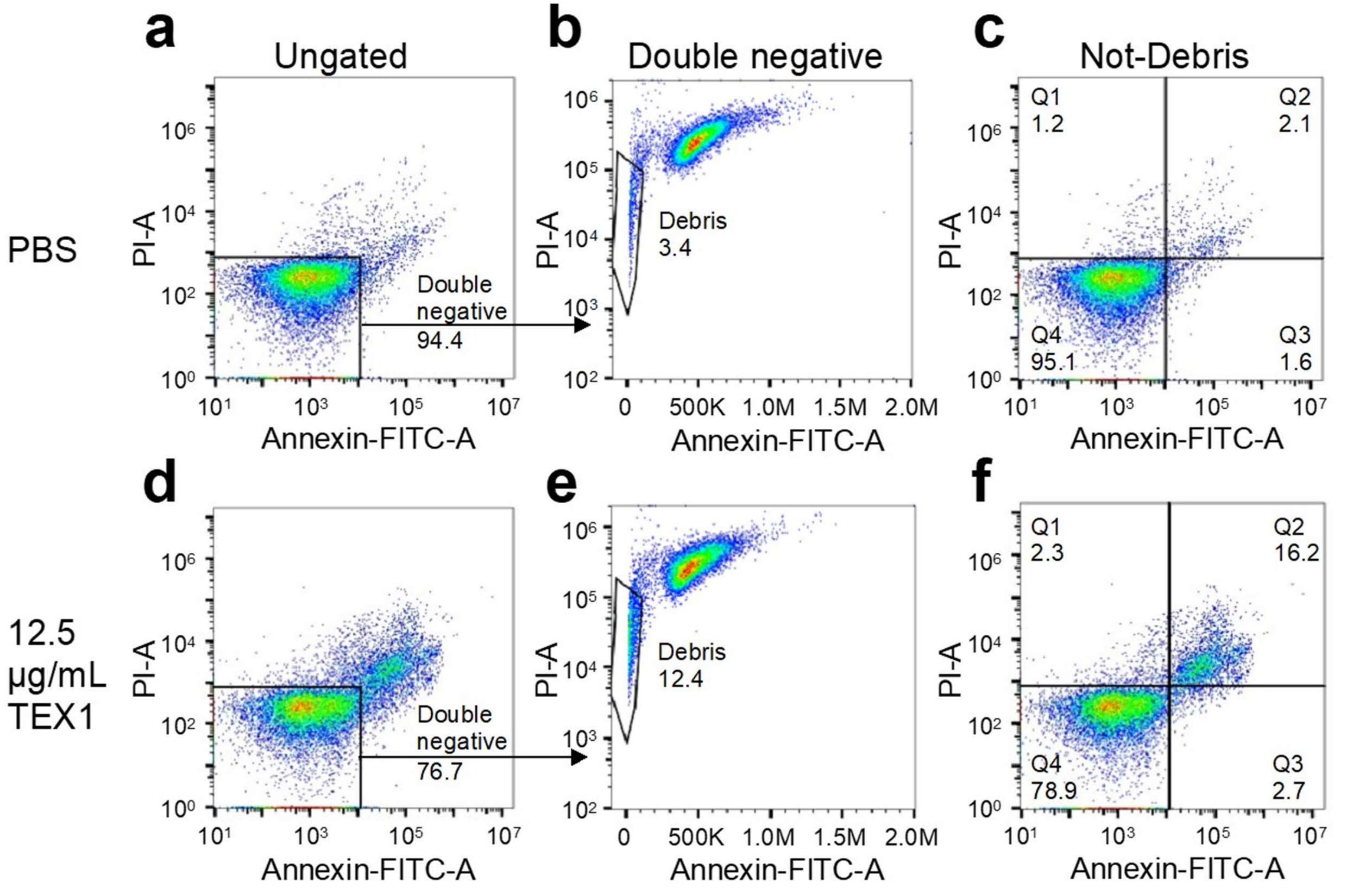

Supplementary Figure 6. Representative gating strategy for PBS-treated (a-c) or TEX1-treated (12.5 µg/mL) CD8+ Jurkat T cells (d-f) illustrating sub-gating on non-debris T cell population. First, plot a was drawn representing all recorded events. A gate was drawn in plot a, selecting Annexin-V-FITC(-)/PE(-) population, labeled as Double negative. In plot b, the gate was drawn which excluded double negative population, and the subsequent analysis was done with the double negative, non-debris fraction, as illustrated in plot c. The same gating strategy was applied to all other apoptosis analyses performed.

Supplementary Table 1: RFI of Immunosuppressive proteins in sEV isolated from HDs or TNBC-Pts plasma

TNBC-Pt	PDL1	PD1	FasL	Fas	Lap-TGFβ	TRAIL	CD39	CD73	CTLA 4	Supp RFI score[a]
**TNBC-Pt #1**	18.32	7.09	20.59	22.60	3.35	9.87	1.14	1.11	4.06	88.14
**TNBC-Pt #2**	12.98	8.00	16.33	13.57	3.60	8.24	1.60	1.35	3.40	69.06
**TNBC-Pt #3**	22.36	5.40	18.47	18.72	2.96	9.82	0.92	1.01	4.36	84.02
**TNBC-Pt #4**	25.23	6.89	22.47	23.06	3.09	11.48	1.12	1.07	6.50	100.91
**TNBC-Pt #5**	6.56	5.39	7.72	13.22	1.58	5.20	3.00	2.51	0.80	45.99
**HD #1**	3.53	2.87	4.50	9.28	0.94	3.36	1.81	1.27	1.15	28.70
**HD #2**	8.72	4.90	11.52	12.22	1.69	5.54	1.08	0.99	2.37	49.03
**HD #3**	10.72	4.14	13.07	10.24	1.29	6.43	1.20	0.88	2.95	50.92
**HD #4**	2.41	3.10	4.54	4.13	1.68	2.33	0.95	0.93	2.31	22.36
**HD #5**	4.42	2.65	4.01	6.35	1.28	3.57	0.99	0.81	2.45	26.52

[a]Immunosuppressive RFI score.

Supplementary Table 2: RFI of Immunostimulatory proteins in sEV from HDs or TNBC-Pts plasma

TNBC-Pt	CD40L	CD40	OX40L	OX40	CD80	stimu RFI score[b]
**TNBC-Pt #1**	158.48	28.04	102.22	2.58	12.22	303.55
**TNBC-Pt #2**	94.26	24.55	73.76	1.21	4.95	198.72
**TNBC-Pt #3**	140.33	24.12	75.85	3.44	7.65	251.40
**TNBC-Pt #4**	161.75	22.54	102.39	1.13	11.15	298.96
**TNBC-Pt #5**	87.66	11.20	53.11	1.26	7.92	161.15
**HD #1**	38.59	5.76	53.08	0.88	5.63	103.94
**HD #2**	77.12	12.38	100.18	1.46	4.52	195.66
**HD #3**	71.61	11.83	96.14	1.39	4.74	185.72
**HD #4**	63.59	10.53	59.17	1.42	4.68	139.41
**HD #5**	78.45	12.31	68.57	1.90	5.82	167.05

[b]Immunostimulatory RFI score.

Supplementary Table 3. Listing of antibodies/reagents used in this study

Antibodies used for western blots of sEV:		
**Name**	**Dilution**	**Manufacturer**
TSG101	1:500	Thermo Fisher, #PA5-31260
CD9	1:500	Thermo Fisher, #10626D
ALIX	1:500	Cell Signaling Technology, #2171S
Calnexin	1:1000	Cell Signaling Technology, #2433
Grp94	1:1000	Cell Signaling Technology, #2104T
Apo B	1:2000	Protein Tech, #20578-I-AP
**Detection antibodies for sEV on bead flow cytometry:**		
PDL-1	1:40	Invitrogen, #12-5983-42
TRAIL	1:40	Invitrogen, #12-9927-42
Fas	1:40	Invitrogen, #12-0959-42
CD40	1:40	Invitrogen, #12-0409-42
OX40	1:40	Invitrogen, #12-1347-42
PD1	1:40	Biolegend, #329904
FasL	1:40	Biolegend, #306407
LAP-TGFβ	1:40	Biolegend, #349606
CD39	1:40	Biolegend, #328206
CD73	1:40	Biolegend, #344016
CD40L	1:40	Biolegend, #310806
OX40L	1:100	Biolegend, #326308
CTLA4	1:40	Pharmingen, #12-1529-42
CD80	1:40	Pharmingen, #557227
**Blocking reagents:**		
Fas Ab	10µg/mL	Millipore, #05-338
anti-PD1 Ab	10µg/mL	R&D Systems, #AF1086
anti-TRAIL Ab	10µg/mL	Thermo Fisher, #16-4714-82
anti-CTLA4 Ab	20µg/mL	BioXcell, #BE0190
TGFβ inhibitor mRER	50nM	Dr. A. Hinck, UPITT
Dynosore	10µM	Selleck Chemicals, #58047
cytochalasin-D	20µM	Tocris, #123
Pit Stop 2	10µM	Millipore Sigma, #SML1169
Abs to human MHC class I	10µg/mL	Dr. S. Ferrone, Harvard U
Abs to human MHC class II	10µg/mL	Dr. S. Ferrone, Harvard U
**Antibodies specific for Survival proteins:**		
BCL-2	1:10	BD Bioscience, #340575
BCL-XL	1:25	Beckman Coulter, #731723
BAX	1:50	Biolegend, #633603
unconjugated anti-cFLIP Abs	1:00	Novus Biologicals, #NBP2-37359

Supplementary Figure 7: Original Western Blots

Raw Western Blot scans for Cell Lines in Figure 1d

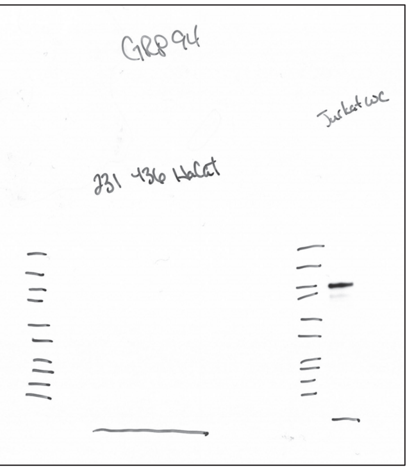

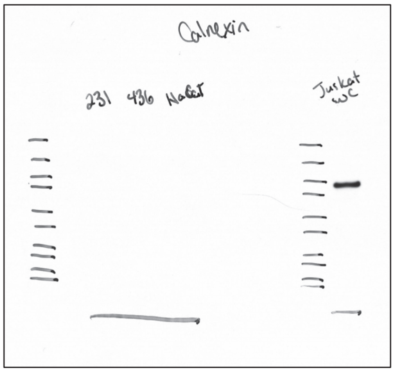

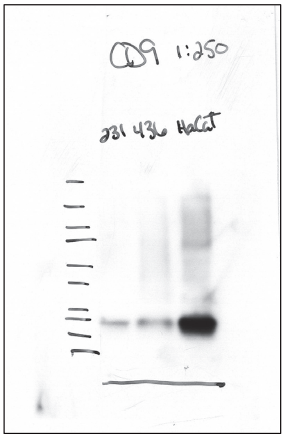

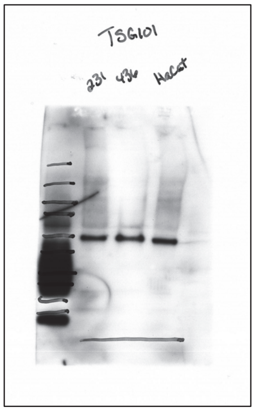

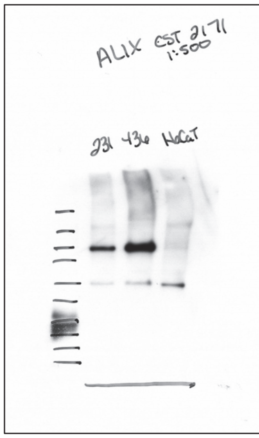

Figure 1d Calnexin

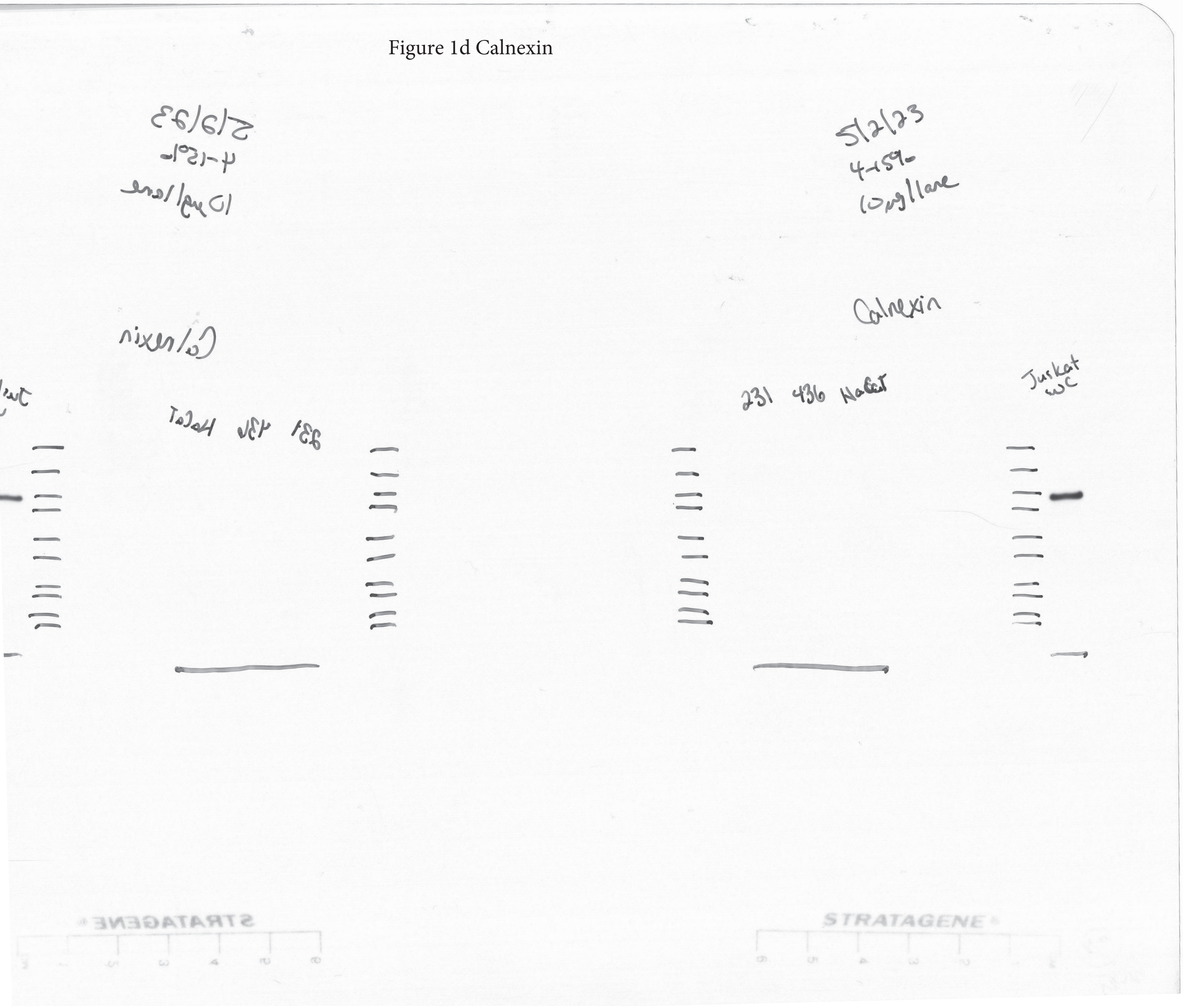

Figure 1d GRP94

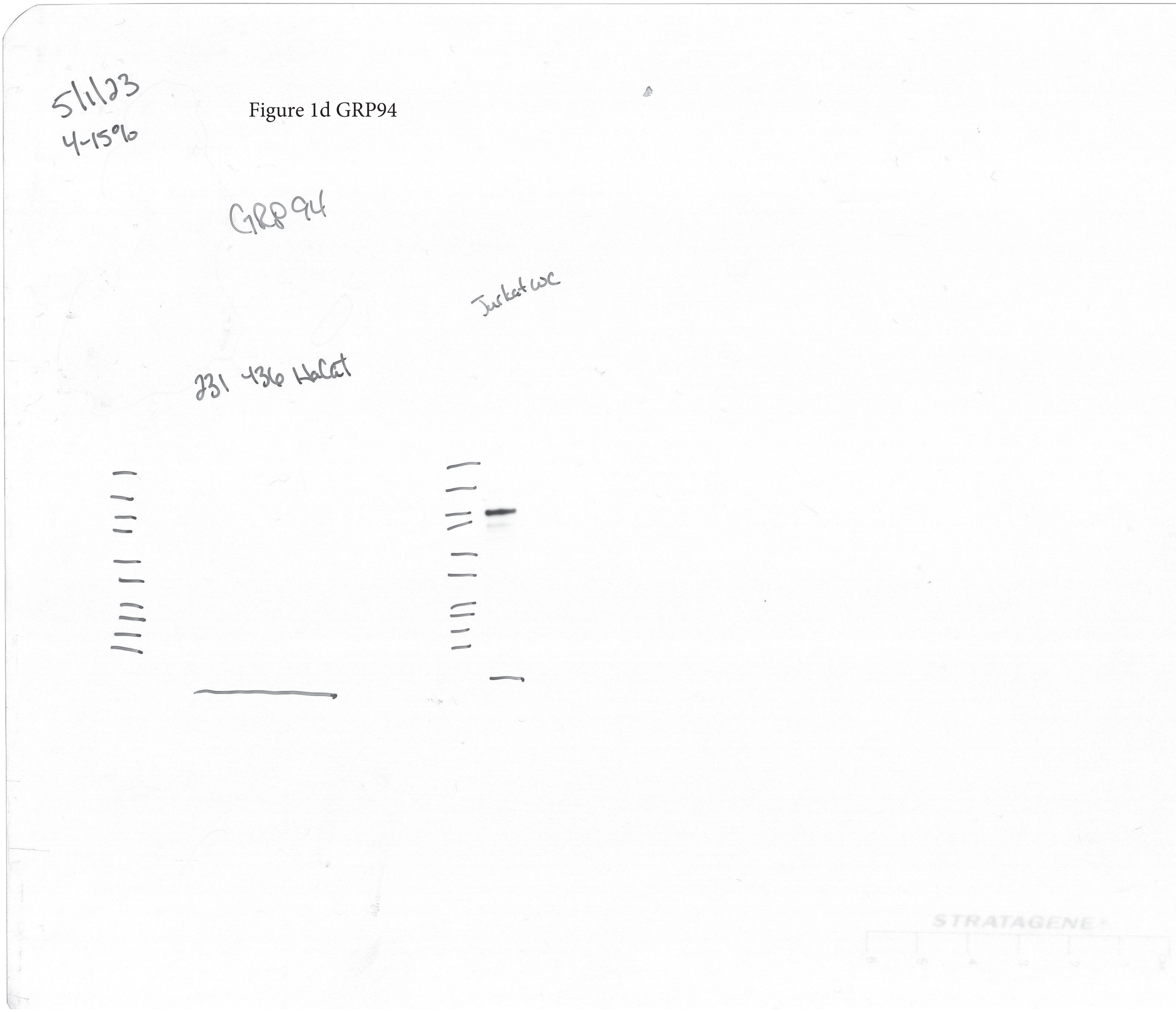

Figure 1d CD9

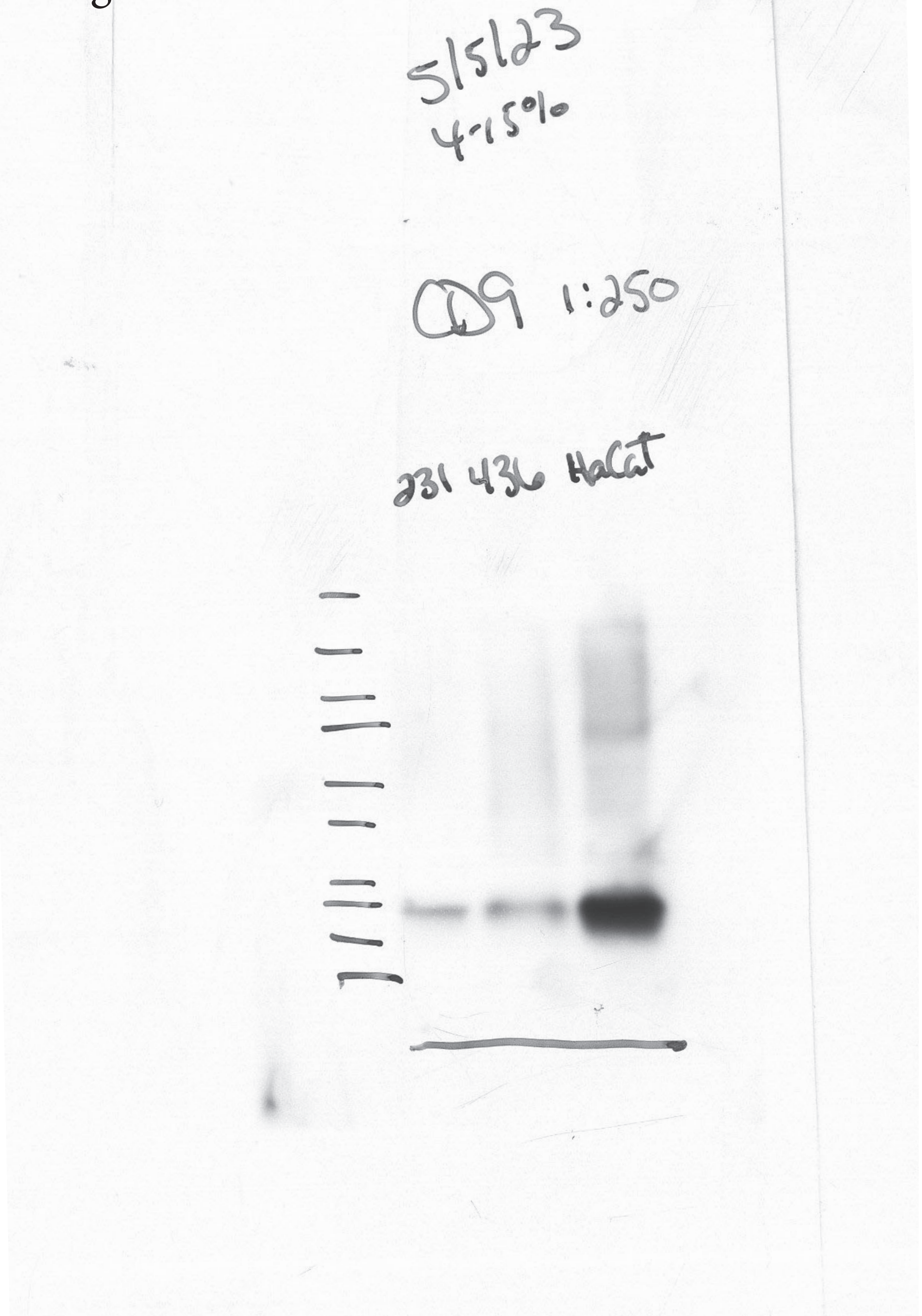

Figure 1d ALIX

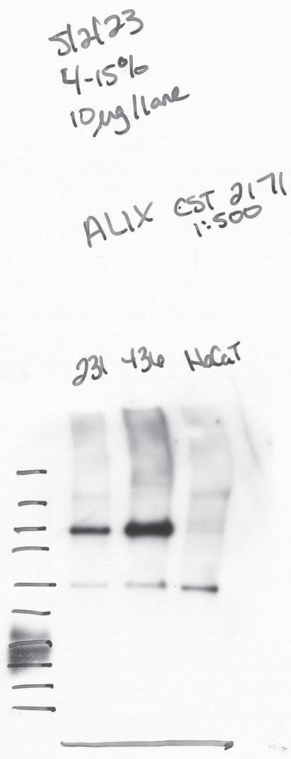

Figure 1d TSG101

5/1/23
4-15% Gel

ALIX

TSG101

231 436 HaCat

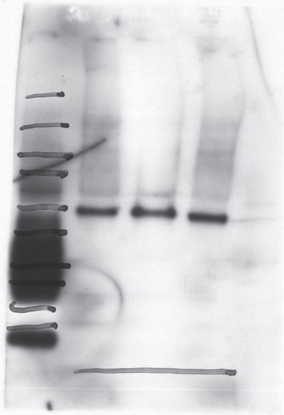

Raw Western Blot scans for Healthy Donor in Figure 2e

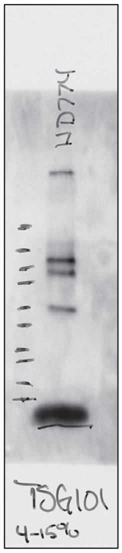

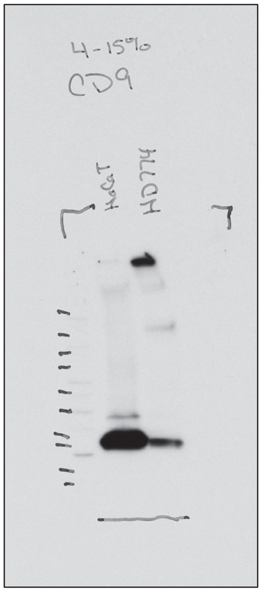

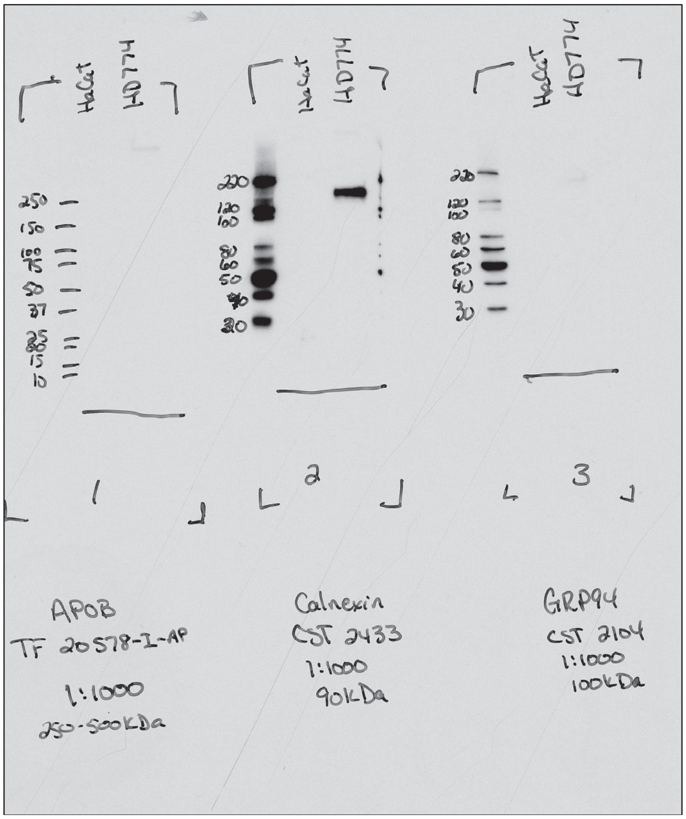

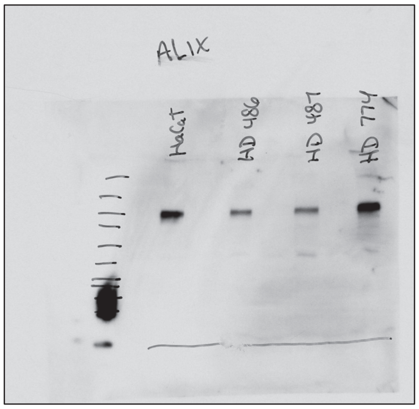

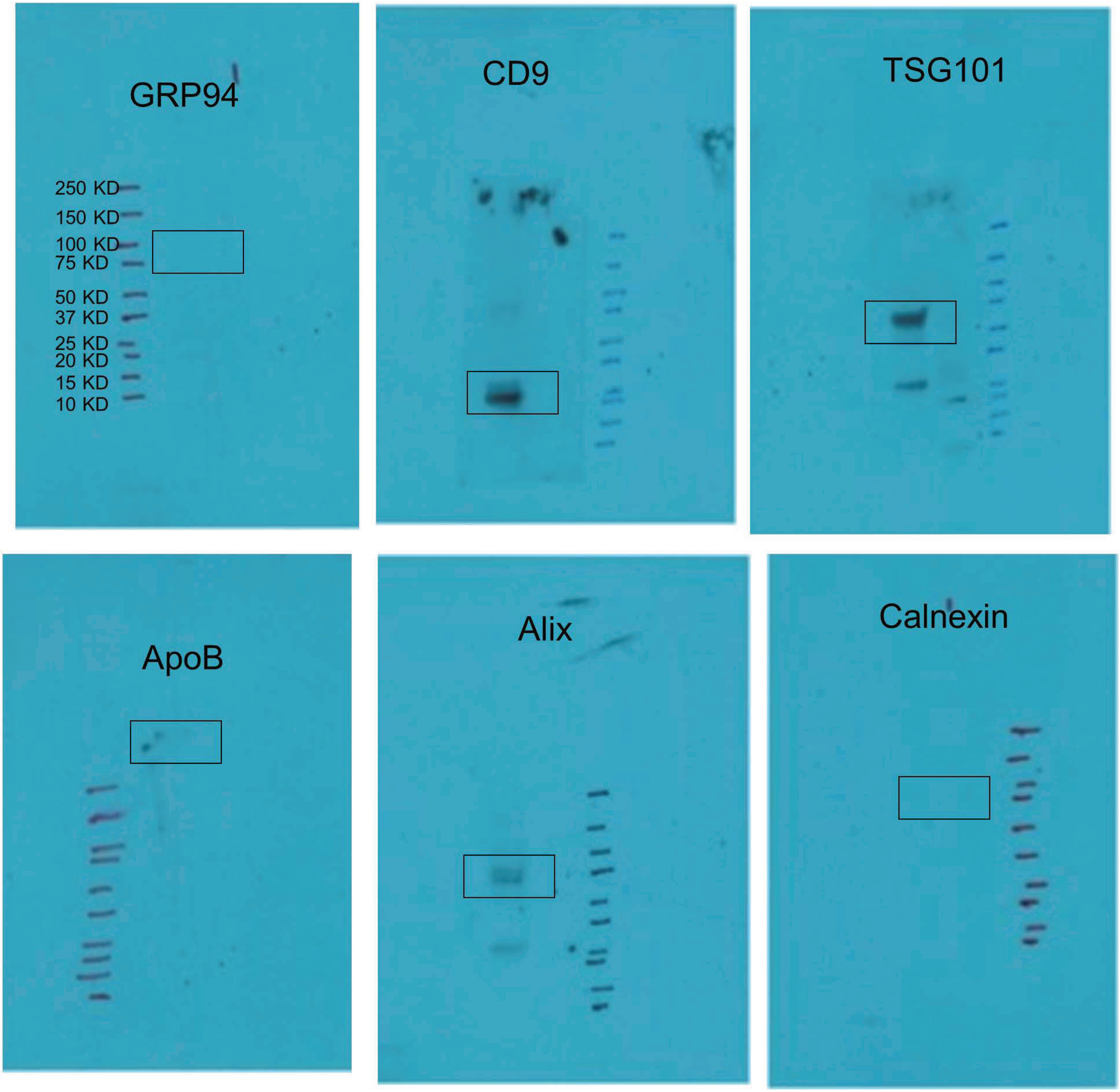
GRP94
250 KD
150 KD
100 KD
75 KD
50 KD
37 KD
25 KD
20 KD
15 KD
10 KD
CD9
TSG101
ApoB
Alix
Calnexin

Image source for S Fig5a

Casp-8

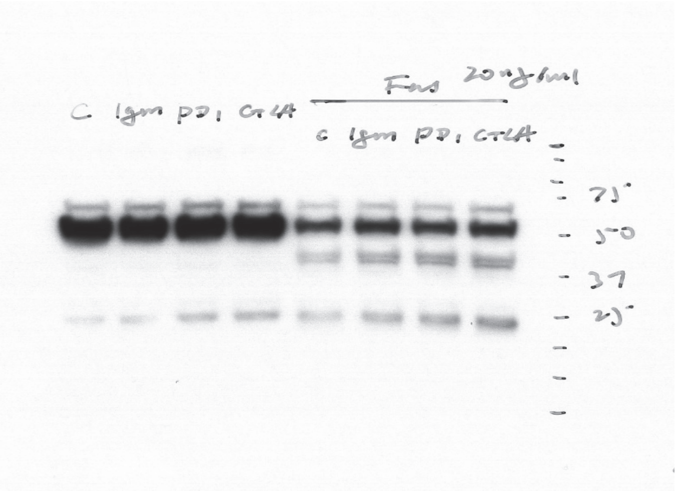

Casp-3

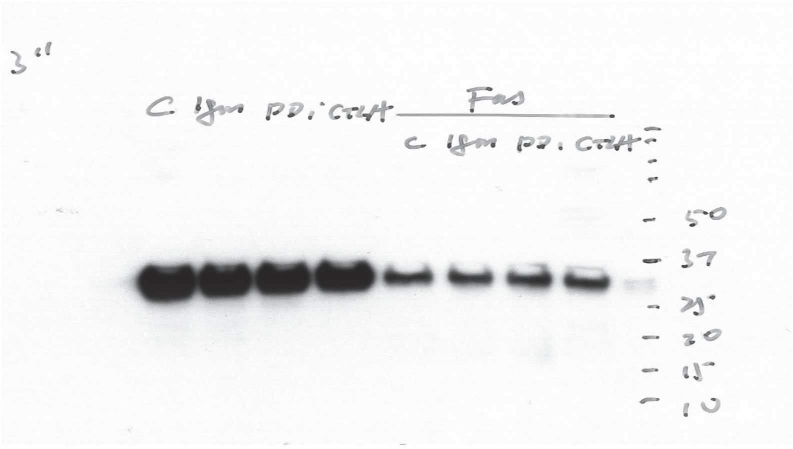

Cleaved
Casp-3

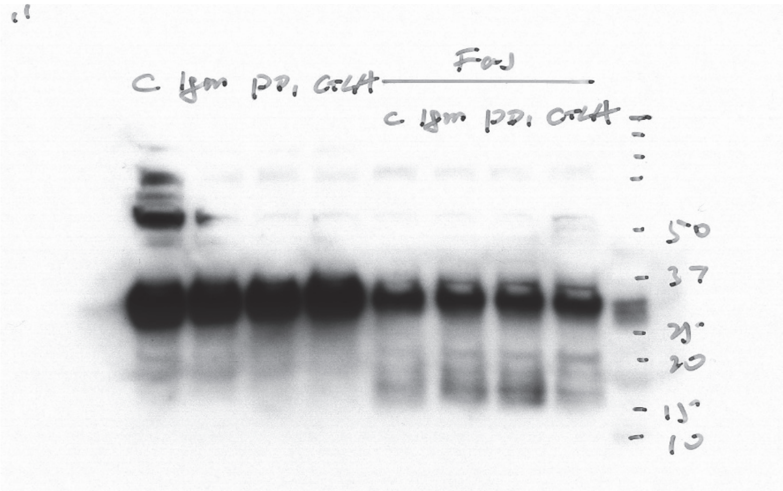

PARP

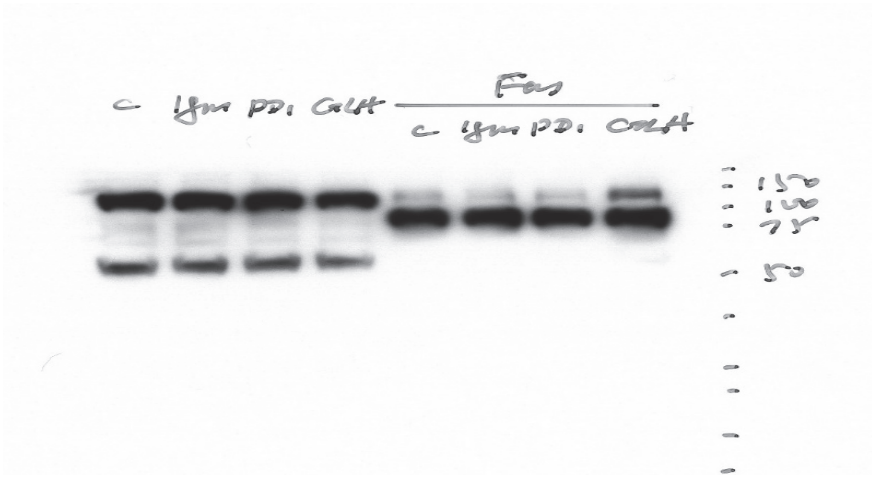

Beta
actin

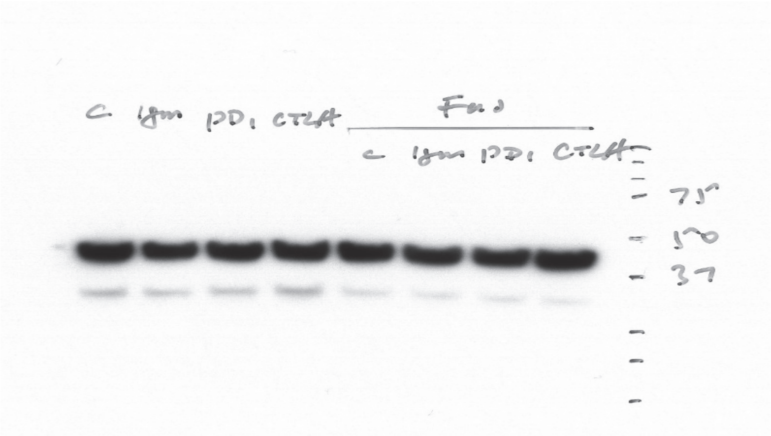

Image source for S Fig5b

Casp-8

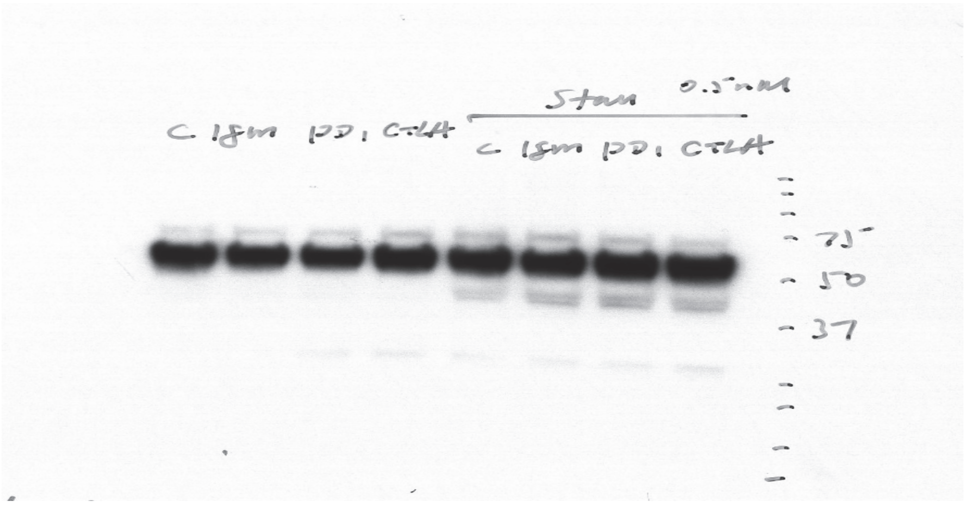

Casp-3

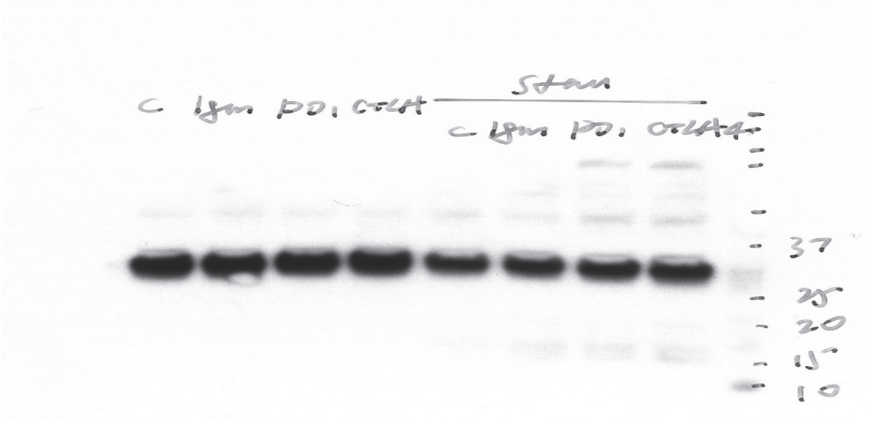

Cleaved
Casp-3

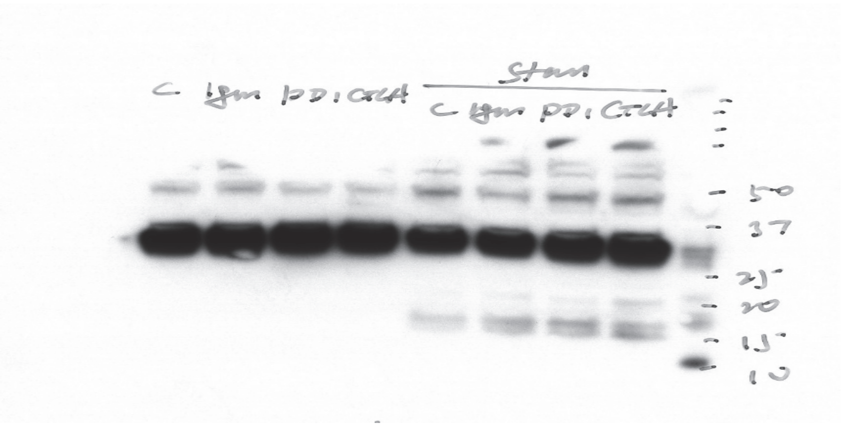

PARP

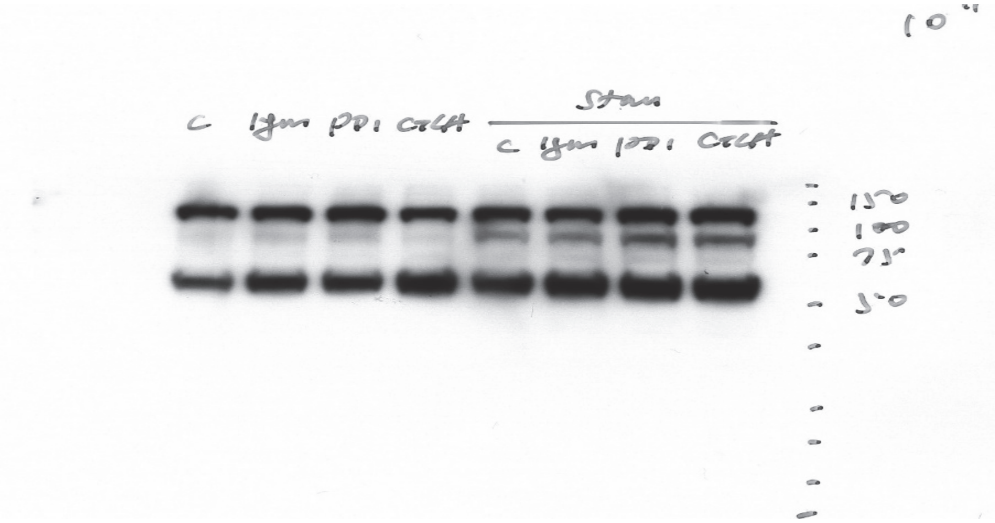

Beta
actin

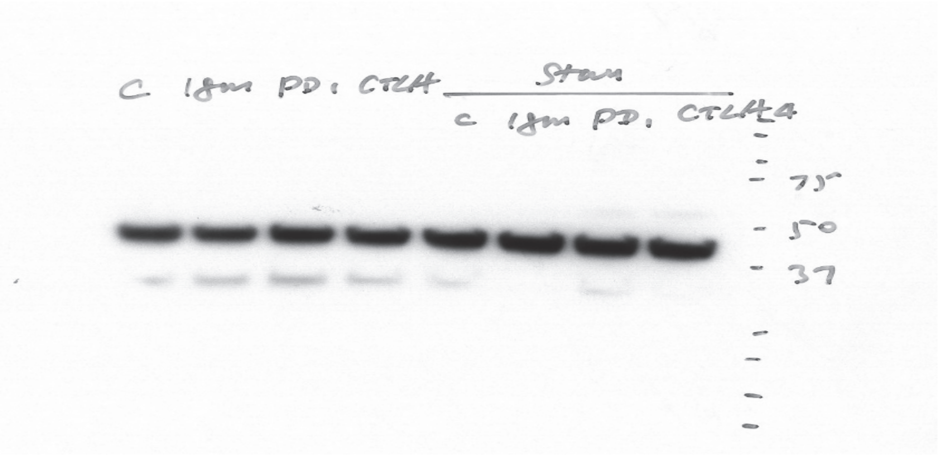

Image source for Fig7e

CytoC

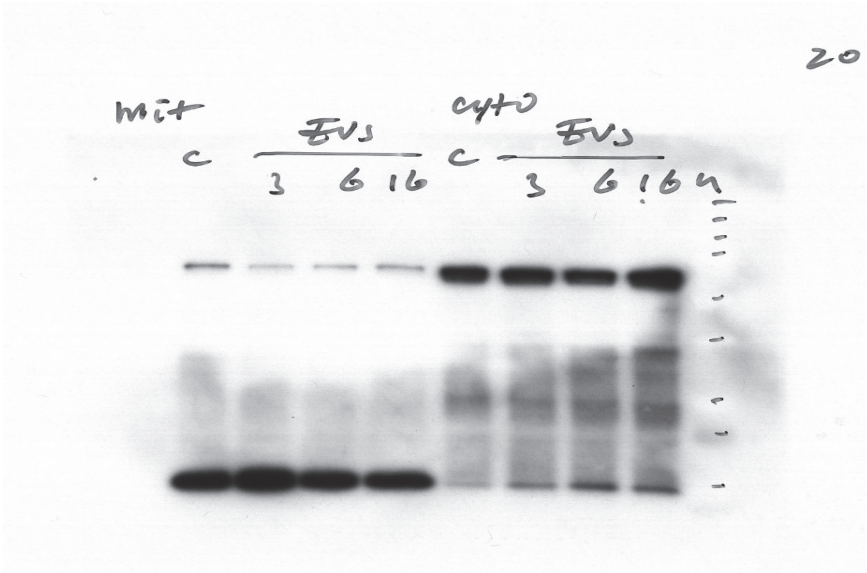

Smac

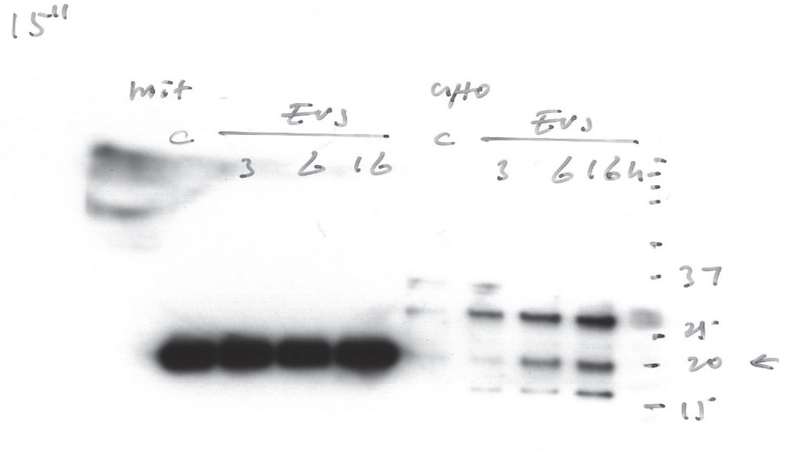

Casp-3
Short
exposure

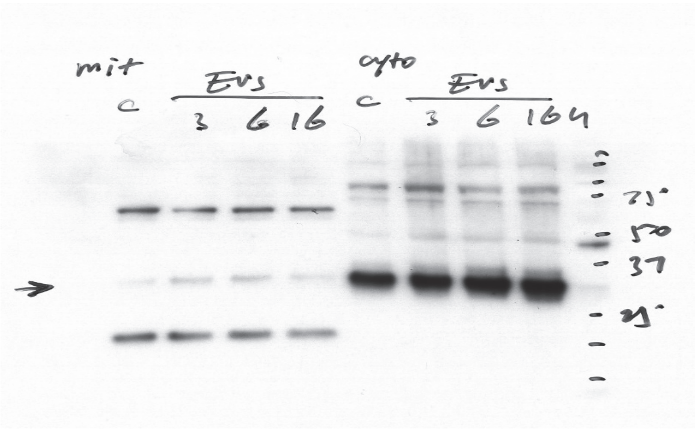

Casp-3
Long
exposure

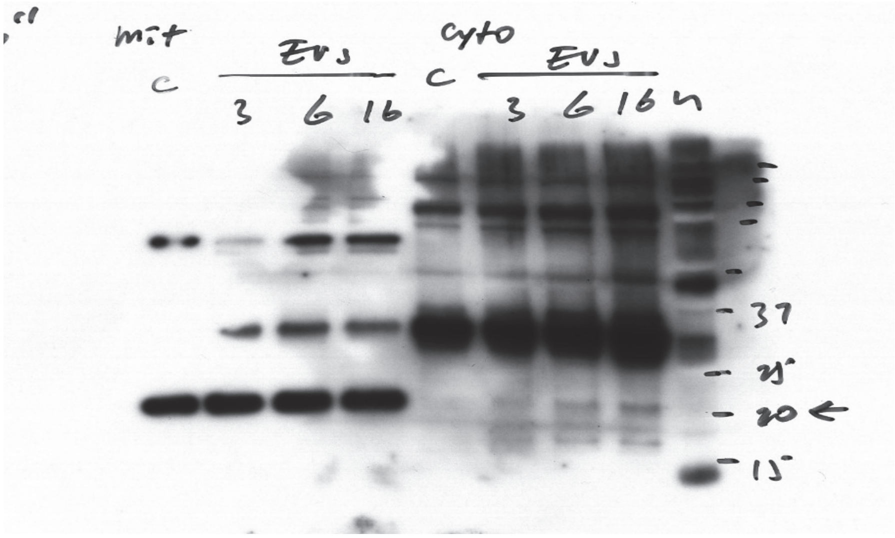

Casp-8

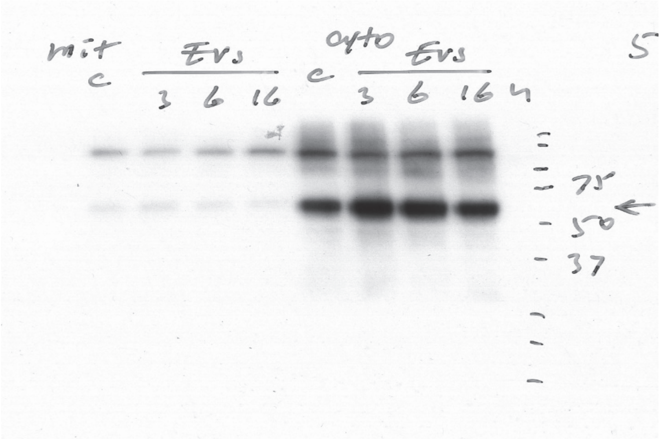

PARP

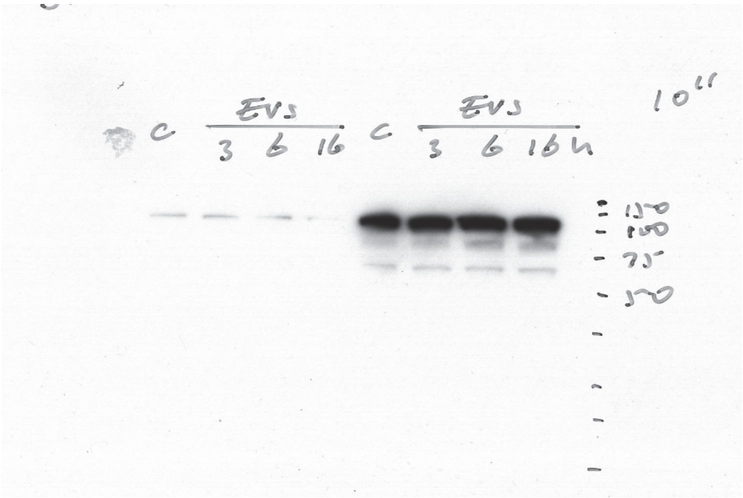

Bcl-xl

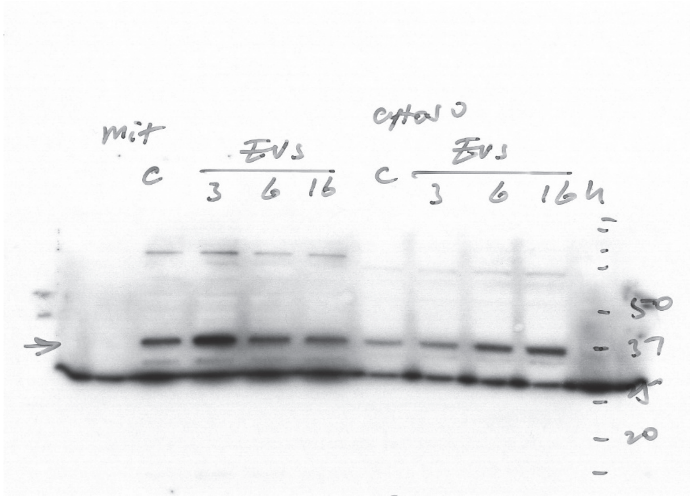

Flip

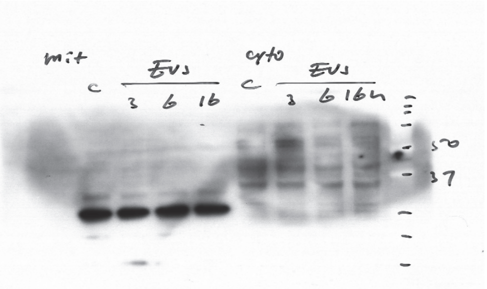

Beta-actin

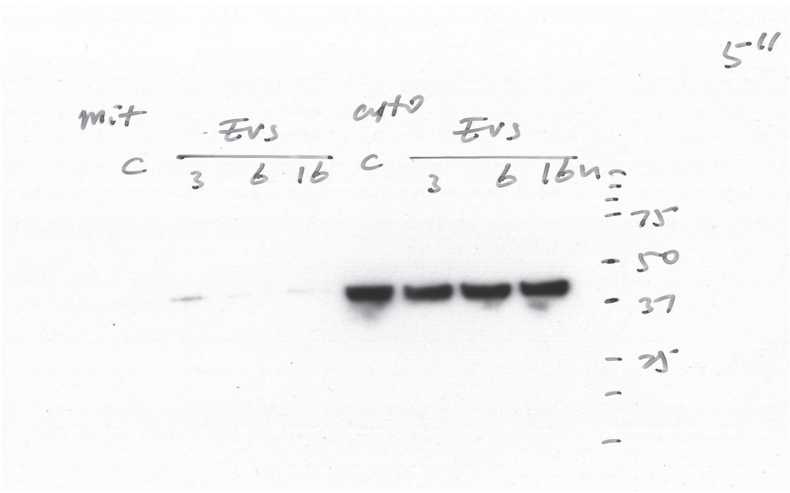

Image source for Fig7d

CytoC

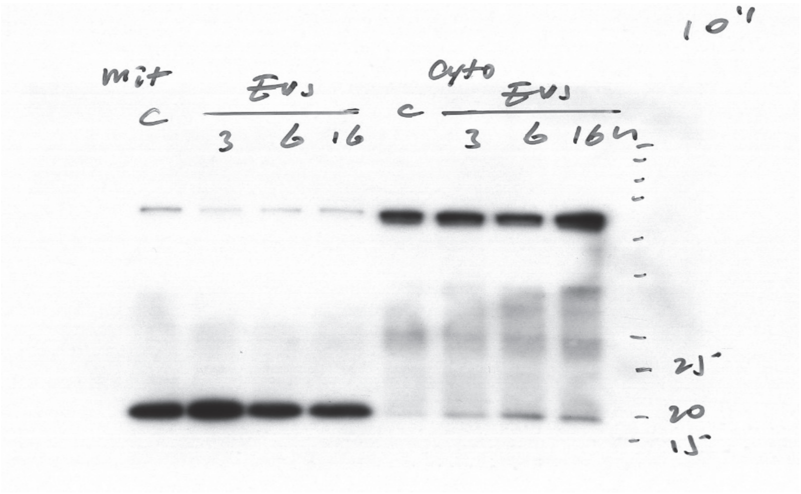

Smac

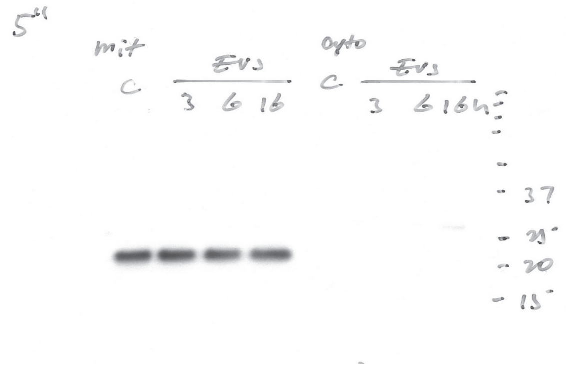

Bcl-2

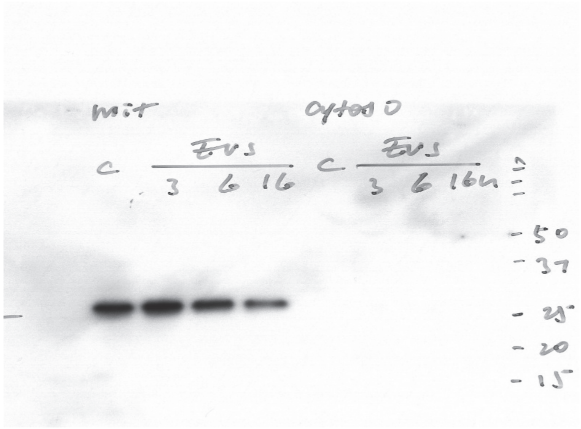

Bcl-xl

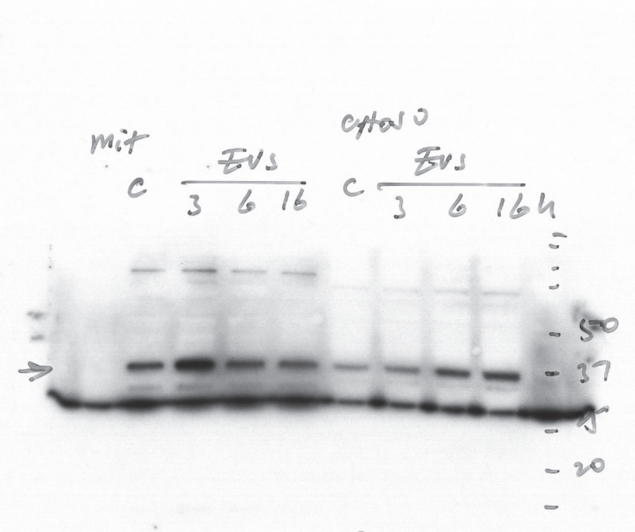

COX-IV

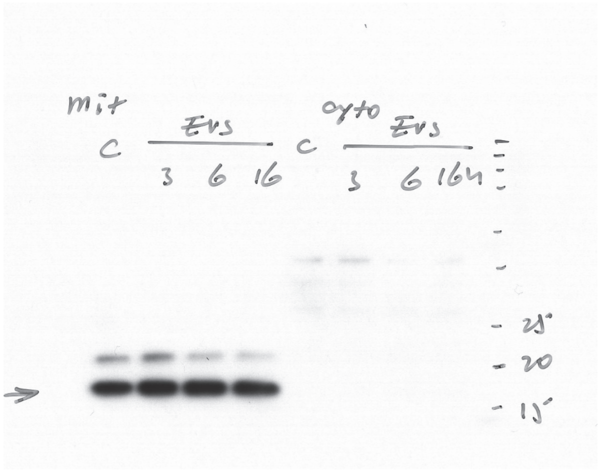

Image source for Fig7f

AIF

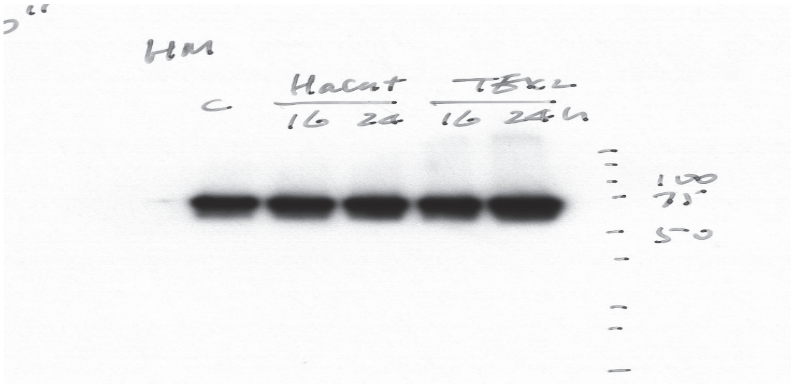

Cyto C

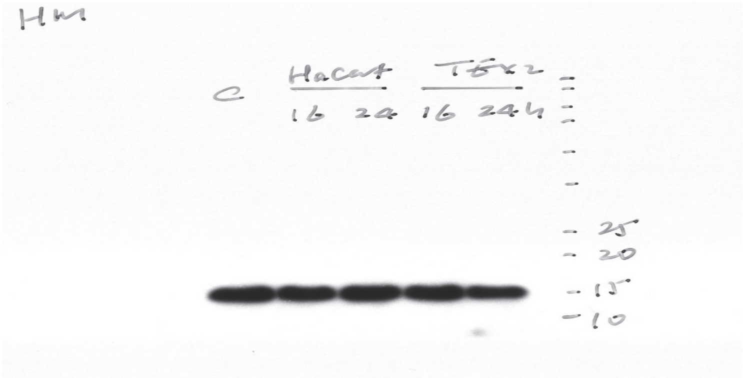

Smac

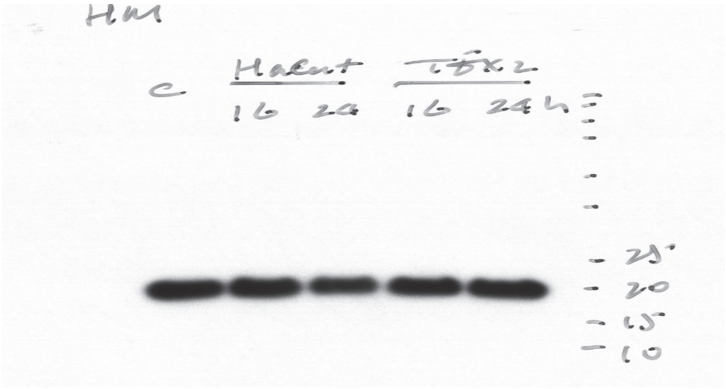

Bcl-2

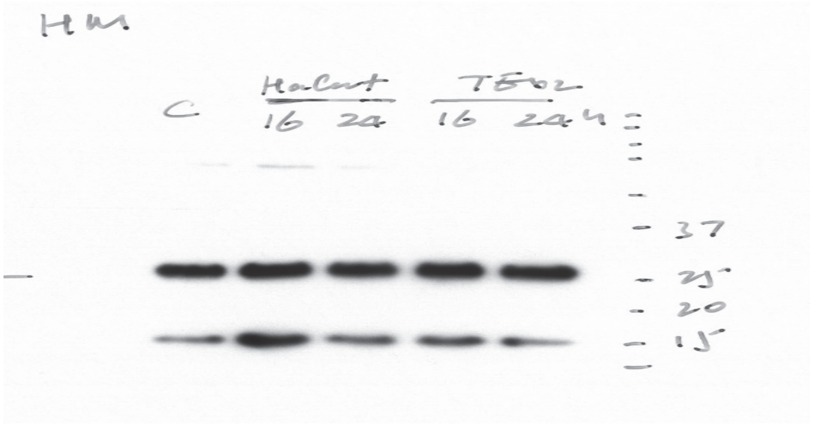

Bcl-xl

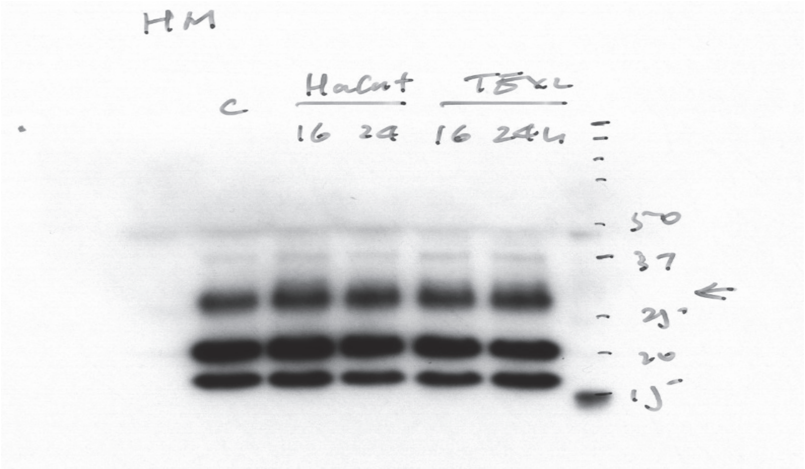

COX IV

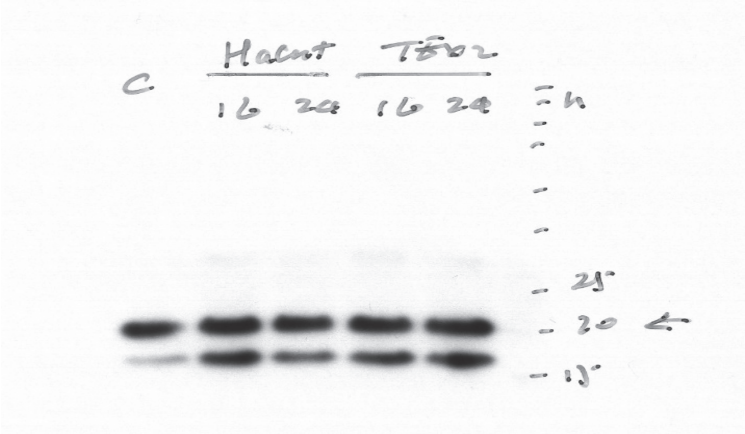

Image source for Fig7g

AIF

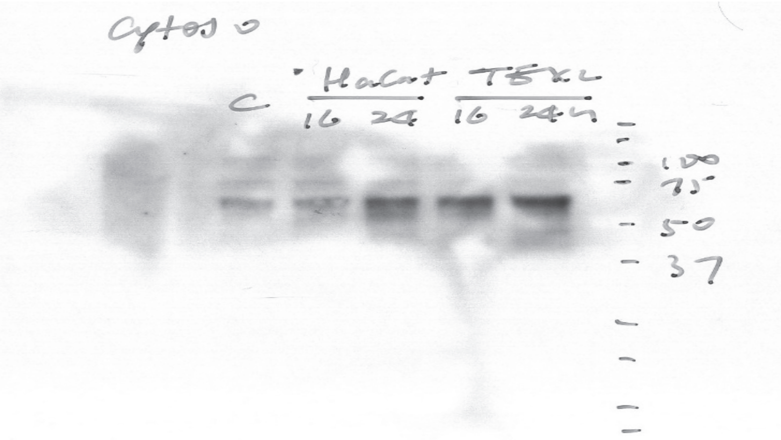

Cyto C

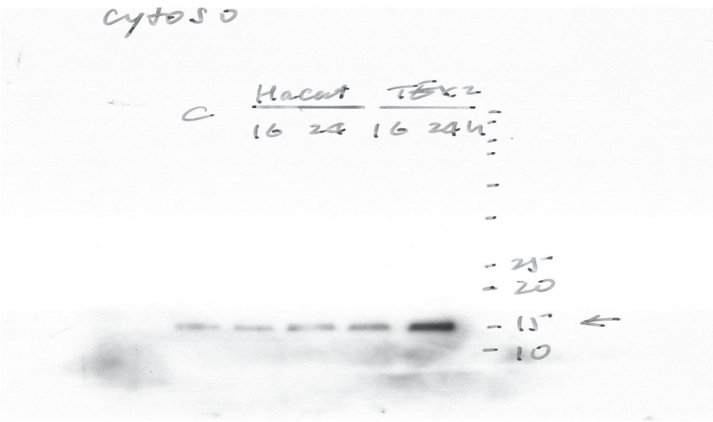

Smac

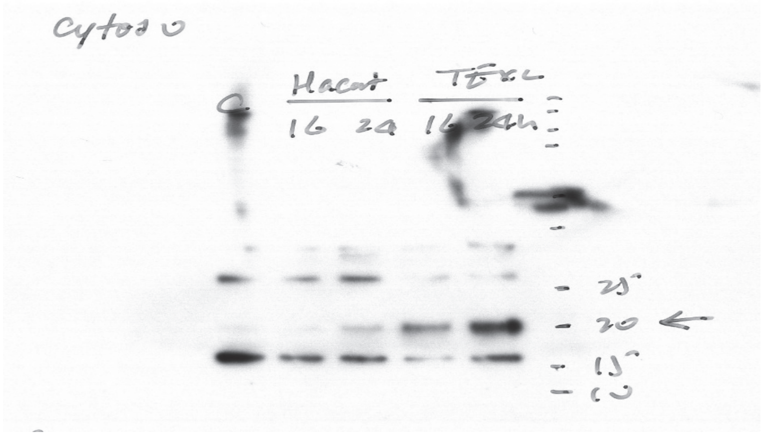

Casp-8

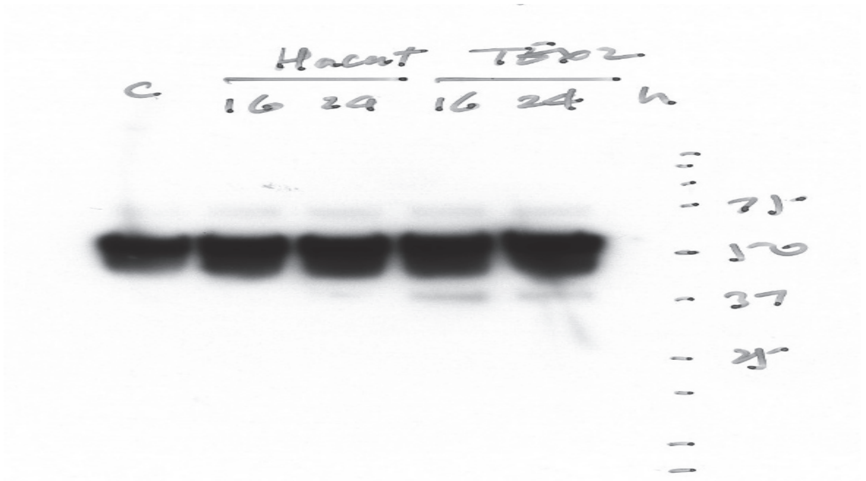

Casp-3

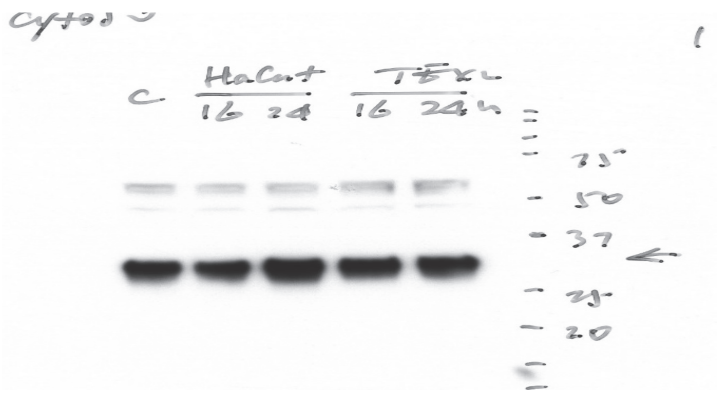

PARP

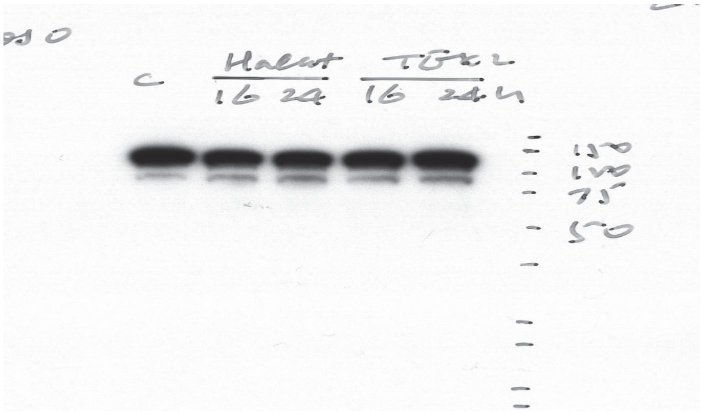

Bcl-xl

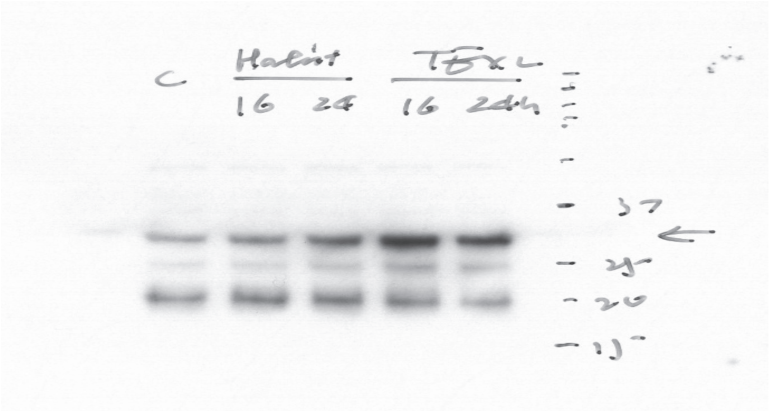

Beta actin

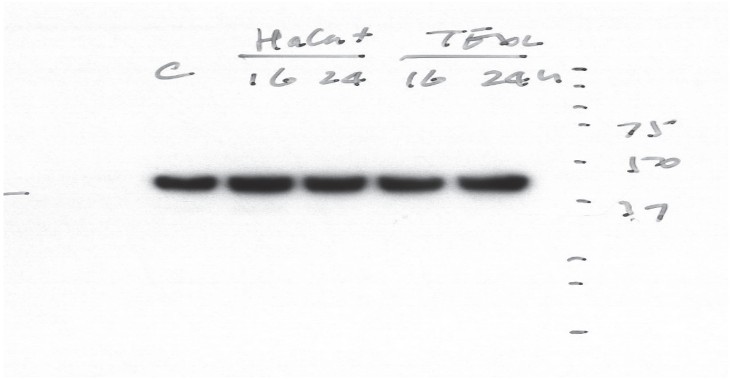

Supplement: Supplementary file 1 — Supplementary Information [file 42003_2023_5169_MOESM1_ESM.pdf]
