# Supplementary material for: Small EV in plasma of triple negative breast cancer patients induce intrinsic apoptosis in activated T cells
Source: Commun Biol. 2023 Aug 4;6:815. doi: 10.1038/s42003-023-05169-3 (PMC10403597; doi:10.1038/s42003-023-05169-3)
Supplement: Supplementary file 2 — Description of Additional Supplementary Files [file 42003_2023_5169_MOESM2_ESM.pdf]

Description of Additional Supplementary Files

File name: Supplementary Data 1

Description: Source data behind the graphs in this manuscript
