# Supplementary material for: Small EV in plasma of triple negative breast cancer patients induce intrinsic apoptosis in activated T cells
Source: Commun Biol. 2023 Aug 4;6:815. doi: 10.1038/s42003-023-05169-3 (PMC10403597; doi:10.1038/s42003-023-05169-3)
Supplement: Supplementary file 4 — Reporting Summary [file 42003_2023_5169_MOESM4_ESM.pdf]

Corresponding author(s): Theresa L. Whiteside
Last updated by author(s): Mar 21, 2023

Reporting Summary

Nature Portfolio wishes to improve the reproducibility of the work that we publish. This form provides structure for consistency and transparency in reporting. For further information on Nature Portfolio policies, see our Editorial Policies and the Editorial Policy Checklist.

Statistics

For all statistical analyses, confirm that the following items are present in the figure legend, table legend, main text, or Methods section.

n/a	Confirmed	
☐	☒	The exact sample size (*n*) for each experimental group/condition, given as a discrete number and unit of measurement
☐	☒	A statement on whether measurements were taken from distinct samples or whether the same sample was measured repeatedly
☐	☒	The statistical test(s) used AND whether they are one- or two-sided<br>*Only common tests should be described solely by name; describe more complex techniques in the Methods section.*
☐	☒	A description of all covariates tested
☐	☒	A description of any assumptions or corrections, such as tests of normality and adjustment for multiple comparisons
☐	☒	A full description of the statistical parameters including central tendency (e.g. means) or other basic estimates (e.g. regression coefficient) AND variation (e.g. standard deviation) or associated estimates of uncertainty (e.g. confidence intervals)
☐	☒	For null hypothesis testing, the test statistic (e.g. *F*, *t*, *r*) with confidence intervals, effect sizes, degrees of freedom and *P* value noted<br>*Give P values as exact values whenever suitable.*
☒	☐	For Bayesian analysis, information on the choice of priors and Markov chain Monte Carlo settings
☒	☐	For hierarchical and complex designs, identification of the appropriate level for tests and full reporting of outcomes
☒	☐	Estimates of effect sizes (e.g. Cohen's *d*, Pearson's *r*), indicating how they were calculated

Our web collection on statistics for biologists contains articles on many of the points above.

Software and code

Policy information about availability of computer code

Data collection	Data have been collected on personal computer using Microsoft Excel protected by a passsword.
Data analysis	Data analysis was performed using Graph Prism 8.3 software on a personal computer protected by a password.

For manuscripts utilizing custom algorithms or software that are central to the research but not yet described in published literature, software must be made available to editors and reviewers. We strongly encourage code deposition in a community repository (e.g. GitHub). See the Nature Portfolio guidelines for submitting code & software for further information.

Data

Policy information about availability of data

All manuscripts must include a data availability statement. This statement should provide the following information, where applicable:

- Accession codes, unique identifiers, or web links for publicly available datasets
- A description of any restrictions on data availability
- For clinical datasets or third party data, please ensure that the statement adheres to our policy

The data sets generated during and/or analysed during the current study are available from the corresponding author on reasonable request. All data generated or analysed during this study are included in this published article (and its supplementary information files).

Human research participants

Policy information about studies involving human research participants and Sex and Gender in Research.

Reporting on sex and gender	*Use the terms sex (biological attribute) and gender (shaped by social and cultural circumstances) carefully in order to avoid confusing both terms. Indicate if findings apply to only one sex or gender; describe whether sex and gender were considered in study design whether sex and/or gender was determined based on self-reporting or assigned and methods used. Provide in the source data disaggregated sex and gender data where this information has been collected, and consent has been obtained for sharing of individual-level data; provide overall numbers in this Reporting Summary. Please state if this information has not been collected. Report sex- and gender-based analyses where performed, justify reasons for lack of sex- and gender-based analysis.*
Population characteristics	*Describe the covariate-relevant population characteristics of the human research participants (e.g. age, genotypic information, past and current diagnosis and treatment categories). If you filled out the behavioural & social sciences study design questions and have nothing to add here, write "See above."*
Recruitment	*Describe how participants were recruited. Outline any potential self-selection bias or other biases that may be present and how these are likely to impact results.*
Ethics oversight	*Identify the organization(s) that approved the study protocol.*

Note that full information on the approval of the study protocol must also be provided in the manuscript.

Field-specific reporting

Please select the one below that is the best fit for your research. If you are not sure, read the appropriate sections before making your selection.

☒ Life sciences ☐ Behavioural & social sciences ☐ Ecological, evolutionary & environmental sciences

For a reference copy of the document with all sections, see nature.com/documents/nr-reporting-summary-flat.pdf

Life sciences study design

All studies must disclose on these points even when the disclosure is negative.

Sample size	All in vitro experiments with cells or cell lines were performed in triplicate .
Data exclusions	In case of one or two outliers in each triplicate experiment, the experiment is repeated.
Replication	The experiment is repeated using the same material and conditions to verify reproducibility.
Randomization	The difference between two groups are measured by two-sided t-test without any prior assumption of treatment effects.
Blinding	In the reported experiments no blinding was used; the identity of samples used was necessary to select the assay parameters

Reporting for specific materials, systems and methods

We require information from authors about some types of materials, experimental systems and methods used in many studies. Here, indicate whether each material, system or method listed is relevant to your study. If you are not sure if a list item applies to your research, read the appropriate section before selecting a response.

Materials & experimental systems

n/a	Involved in the study	
☐	☒	Antibodies
☐	☒	Eukaryotic cell lines
☒	☐	Palaeontology and archaeology
☒	☐	Animals and other organisms
☒	☐	Clinical data
☒	☐	Dual use research of concern

Methods

n/a	Involved in the study	
☒	☐	ChIP-seq
☐	☒	Flow cytometry
☒	☐	MRI-based neuroimaging

Antibodies

Antibodies used	All antibodies used in this studyare listed in STable2 in the manuscript
Validation	All antibodies used were commercial. The Ab dilutions used and the manufacturers are listed in STable 2.

Eukaryotic cell lines

Policy information about cell lines and Sex and Gender in Research

Cell line source(s)	Human metastatic Triple Negative Breast Cancer Cell Lines (TNBC-CL) MDA-MB-231 (CL 1) and MDA-MB-436 (CL 2) as well as a non-malignant cell line, HaCaT (immortalized human keratinocytes), were obtained from ATCC and were cultured in Dulbecco's modified Eagle's medium (Gibco Fisher Scientific).The Jurkat cell line expressing surface CD8 protein was obtained from Dr H. Rabinowich (Department of Pathology, University of Pittsburgh, PA) and cultured in RPMI medium.
Authentication	The TNBC cell lines came from ATCC with the authentification, Jurkat was not authenticated . It was routinely checked for surface expression of CD8, and found to be at least 80% positive.
Mycoplasma contamination	All cell lines were tested for Mycoplasma using MycoAlert detection Kit from Lonza. All cell line are negative .
Commonly misidentified lines (See ICLAC register)	None

Flow Cytometry

Plots

Confirm that:

- [x] The axis labels state the marker and fluorochrome used (e.g. CD4-FITC).
- [x] The axis scales are clearly visible. Include numbers along axes only for bottom left plot of group (a 'group' is an analysis of identical markers).
- [ ] All plots are contour plots with outliers or pseudocolor plots.
- [x] A numerical value for number of cells or percentage (with statistics) is provided.

Methodology

Sample preparation	sEV isolated by SEC from supernatants of cell lines or banked plasma of TNBC patients , Human immune cells were isolated from peripheral blood of HDs by Ficoll- Paque Plus (GE Healthcare Lifesciences) gradients, an immune cells were were isolated from PBMCs using negative selection-based cell isolation kits from Miltenyi, #130-096-495, #136-096-533, Stem Cell Technologies, #19055, and Biolegend #480061, respectively. Cells were stained with unconjugated Abs and then with PE-conjugated goat -anti mouse IgG for 30 min at 4°C. Isotype control Abs were always included. EV or TEX (10µg/100µL PBS) were incubated with a cocktail of biotin-labeled anti-CD63 mAb (0.5µg, Biolegend, #353018) and biotin-labeled anti-CD9 mAb (0.5µg, Biolegend, #349514) for 2h at RT. Next, streptavidin coated magnetic beads (50µL aliquot; ExoCap™, MBL International, Woburn, MA) were added to the vesicle-Ab mixtures and incubated for 2h at RT. The bead/Ab/vesicle complexes were washed with PBS and dispersed in 50µL PBS. For detection of target antigens, 4µL aliquots of the complex were dispersed in 100µL PBS and blocked with 2% mouse serum. The pre-titered labeled detection Abs were added; beads were washed with PBS and diluted in PBS for antigen detection using flow cytometry. Antigen expression levels were measured as relative fluorescence intensity (RFI) calculated as the ratio of Ag RFI/isotype control RFI.
Instrument	Cytoflex (Beckman Coulter) equipped with a violet lazer for measuring nanovesicles
Software	FlowJo software
Cell population abundance	For cells we acquire at least 10,000 cells per sample. For exosomes, which are captures on beads we acquire 10,000 events.
Gating strategy	To identify and enumerate sEV, gates were set using the Di-8 dye and the number of positive vesicles in the gate was determined. To enumerate and study cells, gates were set using SSC and FSC. In both cases, isotype controls were used as a negative signal, and Ab labeled vesicles/cells as a positive signal. The SE index was calculated as the ratio of MFI experimental/MFI control values.

- [x] Tick this box to confirm that a figure exemplifying the gating strategy is provided in the Supplementary Information.
